# Simultaneous Nonmotor Symptoms Do Not Affect General Validity but Interpretation of the Parkinson's Disease Motor Diary

**DOI:** 10.1002/mdc3.70061

**Published:** 2025-04-03

**Authors:** Hampus Andersson, Alexander Bremer, Florin Gandor, Georg Ebersbach, Matthias Löhle, Per Odin, Alexander Storch

**Affiliations:** ^1^ Division of Neurology Department of Clinical Sciences, Lund University Lund Sweden; ^2^ Department of Neurology University of Rostock Rostock Germany; ^3^ Movement Disorder Clinic Beelitz Germany; ^4^ Department of Neurology Otto‐von‐Guericke University Magdeburg Germany; ^5^ Department of Neurology, Rehabilitation Medicine, Memory and Geriatrics Skåne University Hospital Lund Sweden; ^6^ German Center for Neurodegenerative Diseases (DZNE) Rostock‐Greifswald Rostock Germany; ^7^ Present address: Department of Therapy Science I Faculty 4—Institute for Health, Brandenburg University of Technology Senftenberg Germany

**Keywords:** Parkinson's disease, nonmotor symptoms, motor fluctuations, PD home motor diary, validity

## Abstract

**Background:**

Motor fluctuations are routinely documented using the Parkinson's disease (PD) home diary. However, the validity of this diary when compared to clinical observers is limited.

**Objective:**

This study disassembled the effects of nonmotor symptoms (NMS) on inter‐method agreement between the PD home motor diary and clinical observers (outside validity criterion).

**Methods:**

A prospective observational VALIDATE‐PD cohort study in advanced PD assessing symptom severity by simultaneous hourly ratings using the home diary (Off, On, dyskinetic state) and a nonmotor diary (11 key NMSs) was performed. Test validity measures (accuracy and Cohen's κ) were compared between hours with and without co‐occurring NMS.

**Results:**

Four hundred eighty‐seven hourly time periods from 47 participants were analyzed. Inter‐method agreement (accuracy, Cohen's κ) of the motor diary was independent of co‐occurring NMSs, but simultaneous NMSs inversely influence false ratings of motor Off and On states.

**Conclusions:**

Simultaneously occurring NMSs do not affect general validity but the interpretation of the PD home motor diary.

Parkinson's disease (PD) progression is associated with fluctuations in motor function and development of dyskinesia.^1^, ^2^, ^3^, ^4^ Hauser and co‐workers developed the PD home diary to quantify motor fluctuations as patient‐defined outcome measure for clinical trials.^5^, ^6^, ^7^ In this diary, patients are asked to indicate their predominant status during half‐hour time periods throughout the day using the categories asleep, Off, On without dyskinesias, On with nontroublesome dyskinesia, and On with troublesome dyskinesia. The diary allows the calculation of daily Off time and daily On time with and without troublesome dyskinesia, which can then be used as outcome variables to assess the effects of interventions in advanced PD.^8^, ^9^, ^10^, ^11^, ^12^, ^13^ Despite its widespread use, the diary has been validated only against direct clinical observation recently in the VALIDATE‐PD study.^14^, ^15^ These data have demonstrated that inter‐method agreement between professional observers and patients using the PD home diary is fair only with a Cohen's κ of 0.358 to 0.387.^14^, ^15^, ^16^ The reasons for the limited validity remain unclear, but simultaneously occurring nonmotor symptoms (NMS) and/or its fluctuations^17^, ^18^, ^19^ are candidate confounders. Indeed, our previous report found on the patient level that depression and cognitive dysfunction are independent predictors of PD home diary agreement with clinical observer ratings.^15^ We here use the VALIDATE‐PD data on the hour time level^14^, ^15^ complemented by simultaneous diary‐based NMS assessment^20^, ^21^ to disassemble the effects of NMSs on inter‐method agreement between the PD home motor diary and simultaneous motor rating by clinical observers.

## Patients and Methods

### Study Protocol

Data reported were obtained from the German cohort of the prospective observational cohort of the VALIDATE‐PD study.^14^, ^15^ For more details on study protocol and baseline assessment, refer to Supplementary Methods in Data S1. All participants received detailed instructions and watched a training video to record their experienced motor state on an hourly basis in the PD home diary for 1 day (Supplementary Methods in Data S1).^5^, ^6^, ^7^, ^15^ The motor diary used the categorization of motor states originally put forth by Hauser and co‐workers^6^: asleep, Off state, On without dyskinesia (On state), On with nontroublesome dyskinesia, and On with troublesome dyskinesia (combined as dyskinetic state). Participants were simultaneously tasked with hourly rating NMS as present or absent during waking hours using the NMS diary developed by Ossig and co‐workers based on validated questions.^20^ Eleven NMSs were included: 5 psychiatric NMSs (anxiety, depressive mood, inner restlessness, difficulties with concentration/attention, and hallucinations), 4 autonomic NMSs (excessive sweating, sialorrhea/drooling, bladder urgency, and dizziness), pain, and fatigue. Prior to conducting NMS diary assessments, participants were trained in the definitions of the various NMS states.

Participants were simultaneously assessed by a clinical observer (A.B.) during active motor performance over the course of the 7‐meter Timed‐Up‐and‐Go‐Test (7 m‐TUGT).^22^, ^23^ This experienced physiotherapist had received training in the identification of fluctuating motor states and is a Movement Disorder Society–certified Unified Parkinson's Disease Rating Scale rater. Observations were made during daytime (8:00 a.m. through 6:00 p.m.) on an hourly basis using the same categories as the PD home diary.^15^

### Data Analyses

Statistical analyses were performed using IBM SPSS Statistics, version 28 (IBM Corporation, New York, USA), except for 2 × 2 contingency tables used for the calculation of test validity measures (Microsoft Excel, https://office.microsoft.com/excel). Values are presented as numbers (percentages) or median (interquartile range, IQR). *P* < 0.05 was considered statistically significant. Due to the explorative character of the study, *P*‐values were not adjusted for α inflation. Handling of missing or inconclusive data is described in Supplementary Methods in Data S1.

Test validity measures for the detection of the 3 observer‐rated motor states by the simultaneous hourly ratings of the patient‐rated PD home diary were calculated using the 2 × 2 contingency matrix for the 3 different motor states separately. The following test performance measures were determined (see Supplementary Methods in Data S1 for additional measures): accuracy in percentage,^24^ sensitivity (or recall) and specificity in percentage, and false‐positive/false‐negative rate (FPR/FNR) in percentage and Cohen's κ.^25^ By translating diary data of all motor states into a 2 × 2 contingency matrix (correct/not correct answers), we calculated the overall accuracy for all motor states together. Multivariate binary logistic regression analyses were performed to determine the effects of 7 m‐TUGT times and NMS diary ratings on the likelihood of Off ratings in both diaries. Further details on statistical methods are presented in Supplementary Methods in Data S1.

## Results

### Demographic and Clinical Data

The final study cohort consisted of 47 participants (see Supplementary Methods in Data S1 for recruitment process) and included 24 (51%) male and 23 (49%) female participants with a median age of 65 (IQR: 58–73). Participants had been diagnosed with PD ~10 years (IQR: 8–15 years) prior to the study and were suffering from motor fluctuations for 61 (IQR: 34–106) months. For further details on study cohort and diary adherence, see Supplementary Results, Tables S1 and S2.

### Overall Accuracy of the PD Home Motor Diary Depending on Simultaneous NMSs

**FIG. 1:**
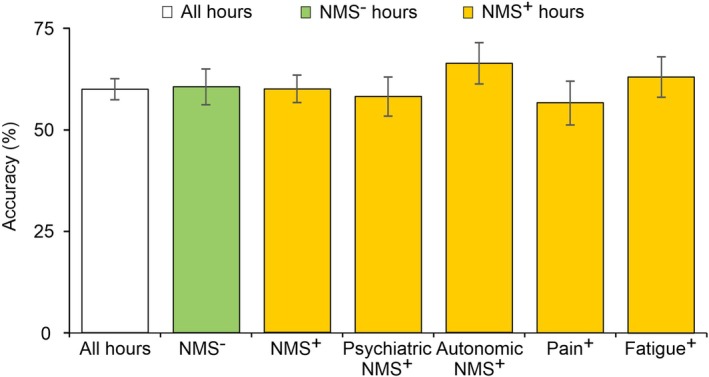
Overall accuracies of the PD (Parkinson's disease) home motor diary compared to observer ratings depending on simultaneous NMS (nonmotor symptom) occurrence. Diagram shows the overall accuracies of the PD home diary in detecting the correct motor states reported by the professional observer PD diary. Values are presented as percentages with interquartile range (IQR) bars. IQR values were calculated using the Clopper–Pearson exact approximation. Accuracy values are reported for all hourly time periods, all hourly time periods with the absence of NMS (NMS^−^), all hourly time periods with presence of NMS (NMS^+^), all hourly time periods with psychiatric NMS present (psychiatric NMS^+^), all hourly time periods with autonomic NMS present (autonomic NMS^+^), all hourly time periods with pain present (Pain^+^), and all hourly time periods with fatigue present (Fatigue^+^). We did not detect any differences in the accuracies between the various NMS^+^ hours and the corresponding NMS^−^ hours for all comparisons with *P* ≥ 0.05 (Pearson's *χ* ^2^ tests, unadjusted for α inflation).

Accuracy as an important test performance measure can by calculated for the overall test performance of the PD home diary for the detection of all 3 motor states together. Figure 1 shows that these accuracy values were almost identical for hours with absent and present NMSs at 61% (IQR: 56%–65%) and 60% (IQR: 57%–63%), respectively. Overall PD home diary accuracies for all NMSs as well as the aggregated NMSs (psychiatric, autonomic NMS), pain, and fatigue are presented in Figure 1. Diary accuracies were independent of sex (Supplementary Results, Fig. S1).

### PD Home Motor Diary Performance for the Different Motor States

**FIG. 2:**
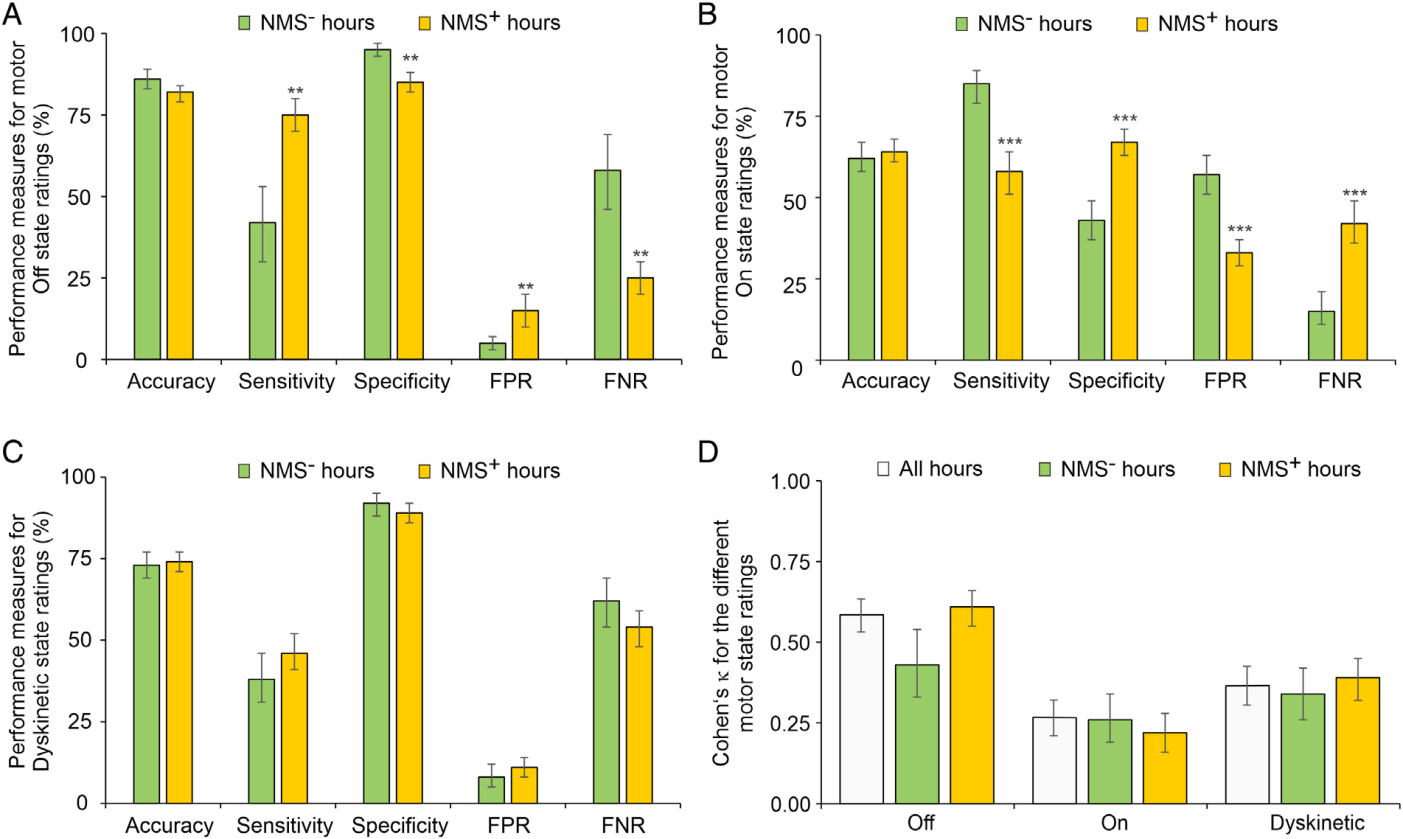
Performance measures for PD (Parkinson's disease) home diary ratings depending on simultaneously occurring NMSs (nonmotor symptoms) with respect to the different motor states. Bar charts comparing the performance measures accuracy, sensitivity, specificity, FPR (false‐positive rate), and FNR (false‐negative rate) during NMS^−^ and NMS^+^ hourly time periods. (**A**) Performance measures for home diary–rated Off hours. (**B**) Performance measures for home diary–rated On hours. (**C**) Performance measures for home diary–rated dyskinetic hours. (A–C) **P < 0.01 and ***P < 0.001 as determined by Pearson's χ^2^ tests. (A–C) Values are presented as percentages, with bars representing the interquartile range (IQR) for all performance measures. IQR values were calculated using the Clopper–Pearson exact approximation. (**D**) A bar chart comparing Cohen's κ values for all hourly time periods as well as NMS^−^ and NMS^+^ hourly time periods in motor Off, On, and On with dyskinesia/dyskinetic. Values are presented as κ values, with IQR bars representing inter‐rater agreement. We did not detect any differences in Cohen's κ values between the various NMS^+^ hours and NMS^−^ hours for all comparisons with *P* ≥ 0.05 (Pearson's χ^2^ tests, unadjusted for α inflation).

The main test validity measures for the 3 motor states are shown in Figure 2 (for numerical results, see Tables S3 and S4). All performance measures are presented for each of the 3 motor states: On, Off, and dyskinetic. When NMS^−^ hours were compared with NMS^+^ hours, test validity measures (accuracy, Cohen's κ) were very similar in both NMS conditions for the detection of all 3 motor states. In contrast, sensitivity/FPR of detecting motor Off state by the PD home diary was increased in NMS^+^ hours at the cost of the respective specificity/FNR (Fig. 2). Interestingly, for detecting the motor On state the opposite effects were observed. Diary performance measures for detecting the dyskinetic state were unaffected by the copresence of NMSs. These effects are mainly driven by the co‐occurrence of psychiatric NMSs and fatigue (for data using aggregated psychiatric and autonomic NMSs, fatigue, and pain, as well as NMS burden, see Tables S3 and S4). All validity measures were independent of sex of study participants (Supplementary Results, Fig. S2). Ancillary analyses revealed no relevant influence of data imbalance (Supplementary Methods, Results, Tables S5–S7).

### Relationship between 7 m‐TUGT Results and Motor Off State Diary Ratings

Multivariate binary logistic regression analyses revealed that 7 m‐TUGT times have significant effects on the likelihood of Off ratings in both diaries, but NMS occurrence increases the probability of Off ratings only in the PD home diary (χ^2^ (2) = 84.8, *P* < 0.001; odds ratio [OR] for 7 m‐TUGT: 1.14, 95% confidence interval [CI]: 1.09–1.18, *P* < 0.001; OR for NMS^+^: 3.80, 95% CI: 2.15–6.70, *P* < 0.001) but has no effects on observer‐documented Off ratings (χ^2^ (2) = 140.1, *P* < 0.001; OR for 7 m‐TUGT: 1.26, 95% CI: 1.20–1.32, *P* < 0.001; OR for NMS^+^: 1.94, 95% CI: 0.94–3.39, *P* = 0.056; Supplementary Results, Fig. S3 and Table S8).

## Discussion

The main finding of the present study is that the simultaneous presence of NMSs does not affect general test validity of the PD home diary for detecting all motor states or for detecting the 3 different motor states separately. Importantly, more specific test performance measures differ between NMS^−^ hours and NMS^+^ hours for detecting the motor Off and On states but not for the dyskinetic state. Co‐occurring NMSs lead to higher sensitivity/FPR at the cost of specificity/FNR for detecting the motor Off state, whereas opposite effects of NMSs were observed for detecting the motor On state. Logistic regression models confirmed that NMS occurrence influences PD home diary Off ratings by increasing their probability by a factor of 3.8. There is no indication that sex influences the validity of the PD home diary.

NMS presence yielded an increased FPR in detecting motor Off state hours compared to NMS^−^ hours. These effects are mainly driven by the co‐occurrence of psychiatric NMSs and fatigue and to a lower extent by pain. This could potentially be attributed to NMS presence causing participants to overestimate the number of hours rated Off, confusing the presence of NMSs with the motor Off state. In line with this reasoning, FNR decreased in concordance with the increase in FPR. This view is supported by the observation that for motor On detection the opposite mechanisms very likely explain the decrease in FPR/increase in FNR of the PD home diary ratings. These 2 inverse effects together with the lack of effects of NMS presence in the detection of dyskinetic state hours translate—at least statistically—into the lack of any notable effects of NMS co‐occurrence on the more general validity measures such as accuracy, balanced accuracy, and Cohen's κ. However, the coincidence of both more prevalent and severe NMSs during motor Off state may instead increase patients' abilities to recognize Off.^19^ Independent of the underlying mechanisms, these observations strongly suggest that changes not only in motor but also in NMS presentations influence ratings of motor Off and On state diary ratings by the patients.

Our study has several limitations (for a detailed discussion of study limitations, see Supplementary Discussion in Data S1). First, the study cohort consisted of a rather small‐sized and heterogeneous cohort, which is however similar to larger cohorts investigating NMSs in advanced PD.^19^, ^26^ Second, motor symptoms and NMSs were self‐reported using diaries with the risk of misunderstandings and misinterpretations of both the practicalities of the diary and the definitions of the different motor and nonmotor states. Third, we applied clinical observation of motor performance as the outside validation criterion with the risk of high inter‐rater variability. We therefore used a single‐rater approach with 1 specifically trained and certified clinical observer to exclude potential bias and furthermore validated observer diary responses against 7 m‐TUGT results.^15^ Although ancillary logistic regression analyses revealed the clear effects of motor function (7 m‐TUGT times) but no influence of NMS occurrence on the likelihood of simultaneous observer‐rated motor Off, their limited explanatory power suggests that they explain only an acceptable proportion of variance. Overall, the exact accuracy of the clinical rater's motor assessments is not known. The aforementioned limitations underline the pilot character of our investigations, and further systematic studies on larger cohorts are required.

In conclusion, the overall validity of the PD home diary remains unchanged by simultaneously co‐occurring NMSs. However, the effects of NMSs on FPR/FNR for motor Off and On state detection are important for the interpretation of numerous clinical trials on treatments of motor fluctuations in advanced PD using the motor Off/On diary ratings as primary or secondary outcomes (as examples^8^, ^9^, ^10^, ^11^, ^12^, ^13^). In light of the present results, these motor diary results are confounded by effects of the investigational treatment on co‐occurring NMSs. Because the PD home diary is a patient‐centered outcome, our findings do not diminish the importance but only the correct interpretation of the trial results with respect to the effects of the investigational interventions on motor symptoms versus NMSs.

## Author Roles

(1) Research project: A. Conception, B. Organization, C. Execution; (2) Statistical analysis: A. Design, B. Execution, C. Review and critique; (3) Manuscript preparation: A. Writing of the first draft, B. Review and critique.

H.A.: 1C, 2B, 2C, 3A

A.B.: 1B, 1C, 2B, 2C, 3B

F.G.: 1B, 1C, 2C, 3B

G.E.: 1B, 1C, 2C, 3B

M.L.: 1A, 1B, 1C, 2B, 2C, 3B

P.O.: 1A, 1B, 1C, 2C, 3B

A.S.: 1A, 1B, 2A, 2B, 3A

## Disclosures

**Ethical Compliance Statement:** Institutional review board approvals for both participating centers were obtained by the Institutional Review Board of the University of Rostock Medical Center (registry number: A 2017‐0115) for the Rostock site and by the Institutional Review Board of the State Medical Association Brandenburg (registry number: AS 84(bB)/2018) for the Beelitz‐Heilstätten site. Written informed consent was obtained from all participants before participation. We confirm that we have read the journal's position on issues involved in ethical publication and affirm that this work is consistent with those guidelines.

**Funding Sources and Conflicts of Interest:** H.A. was supported by intramural funding of the United Neuroscience Campus (UNC) Lund‐Rostock (www.unitedneurosciencecampus.com). No other specific funding was received for this work. The other authors declare that there are no conflicts of interest relevant to this work. The corresponding author had the final responsibility for the decision to submit for publication.

**Financial Disclosures for the Previous 12 Months:** H.A. and A.B. have nothing to disclose. F.G. has received honoraria from AbbVie, Bial, Merz, and Stada outside the submitted work. G.E. has received honoraria for advisory boards, consultancies, and presentations from AbbVie Pharma, Bial, Biogen GmbH, Desitin Pharma, Stada Pharma, Neuroderm Inc., Licher GmbH, UCB Pharma, and Zambon Pharma outside of the present work. He has received royalties from Kohlhammer Verlag and Thieme Verlag. M.L. has received honoraria for presentations from Novartis Pharma and Stada Pharma outside of the present study. P.O. has received funding from AbbVie, Lund University Medical Faculty, MultiPark, the Swedish Parkinson Foundation, Health Care Region Skåne, and Åhlens Foundation outside of the present work. He has received honoraria for lectures and expert advice from AbbVie, Bial, Britannia, Ever Pharma, Global Kinetics, Lobsor, Nordic Infucare, Stada, and Zambon outside of the present study. He has received royalties from UNI‐MED Verlag. A.S. has received funding from the Deutsche Forschungsgemeinschaft (German Research Association) and the Helmholtz‐Association outside the present study. He has received honoraria for presentations/advisory boards/consultations from Global Kinetics Corporation, Esteve, Desitin, Lobsor Pharmaceuticals, Stada, Bial, RG Gesellschaft, Zambon, NovoNordisk, and AbbVie outside the present study. He has received royalties from Kohlhammer Verlag and Elsevier Press. He serves as an editorial board member of *Stem Cells International*.

## Supporting information


**Figure S1.** Overall accuracies of the PD (Parkinson's disease) home motor diary compared to observer ratings for the various NMS (nonmotor symptom) hours depending on sex.
**Figure S2.** Cohen's κ values of the PD (Parkinson's disease) home motor diary compared to observer ratings for the various NMS (nonmotor symptom) hours depending on sex.
**Figure S3.** Relationship between times in the 7 m‐TUGT (7‐meter Timed‐Up‐and‐Go‐Test) and diary Off ratings as documented by participants or observers.

**Table S1.** Demographic and clinical characteristics of study cohort.
**Table S2.** Diary data of waking day hours.
**Table S3.** PD (Parkinson's disease) home motor diary test performance measures depending an NMS co‐occurence from 2 × 2 contingency tables for the detection of clinical observer ratings.
**Table S4.** PD (Parkinson's disease) home motor diary test performance measures depending on NMS burden from 2 × 2 contingency tables for the detection of clinical observer ratings.
**Table S5.** Validity parameters of the PD (Parkinson's disease) home diary for the detection of clinical observer Off ratings in balanced datasets.
**Table S6.** Validity parameters of the PD (Parkinson's disease) home diary for the detection of clinical observer On ratings in balanced datasets.
**Table S7.** Validity parameters of the PD (Parkinson's disease) home diary for the detection of observer dyskinetic state ratings in balanced datasets.
**Table S8.** Effects of NMS (nonmotor symptom) occurrence and times in the 7 m‐TUGT (7‐meter Timed‐Up‐and‐Go‐Test) on likelihood of motor Off state ratings.

**Data S1.** Supporting information.

## Data Availability

The data that support the findings of this study are available on request from the corresponding author. The data are not publicly available due to privacy or ethical restrictions.
