# Supplementary material for: Simultaneous Nonmotor Symptoms Do Not Affect General Validity but Interpretation of the Parkinson's Disease Motor Diary
Source: Mov Disord Clin Pract. 2025 Apr 3;12(7):979–84. doi: 10.1002/mdc3.70061 (PMC12275004; doi:10.1002/mdc3.70061)
Supplement: Supplementary file 1 — Figure S1. Overall accuracies of the PD (Parkinson's disease) home motor diary compared to observer ratings for the various NMS (nonmotor symptom) hours depending on sex. Figure S2. Cohen's κ values of the PD (Parkinson's disease) home motor diary compared to observer ratings for the various NMS (nonmotor symptom) hours depending on sex. Figure S3. Relationship between times in the 7 m‐TUGT (7‐meter Timed‐Up‐and‐Go‐Test) and diary Off ratings as documented by participants or observers. [file MDC3-12-979-s002.docx]

**Supplementary Information – Figures**

**Simultaneous non-motor symptoms do not affect general validity but interpretation of the Parkinson’s Disease motor diary**

**Hampus Andersson, Alexander Bremer, Florin Gandor, Georg Ebersbach, Matthias Löhle, Per Odin, and Alexander Storch**

**Supplementary Figures:**

- **Supplementary Figure S1.** Overall accuracies of the PD Home motor diary when compared to observer ratings for the various NMS hours in dependency of sex.
- **Supplementary Figure S2.** Cohen’s κ values of the PD Home motor diary when compared to observer ratings for the various NMS hours in dependency of sex.
- **Supplementary Figure S3.** Relationship between times in the 7m-TUGT and diary Off ratings as documented by participants or observers.

**Supplementary Figure S1**


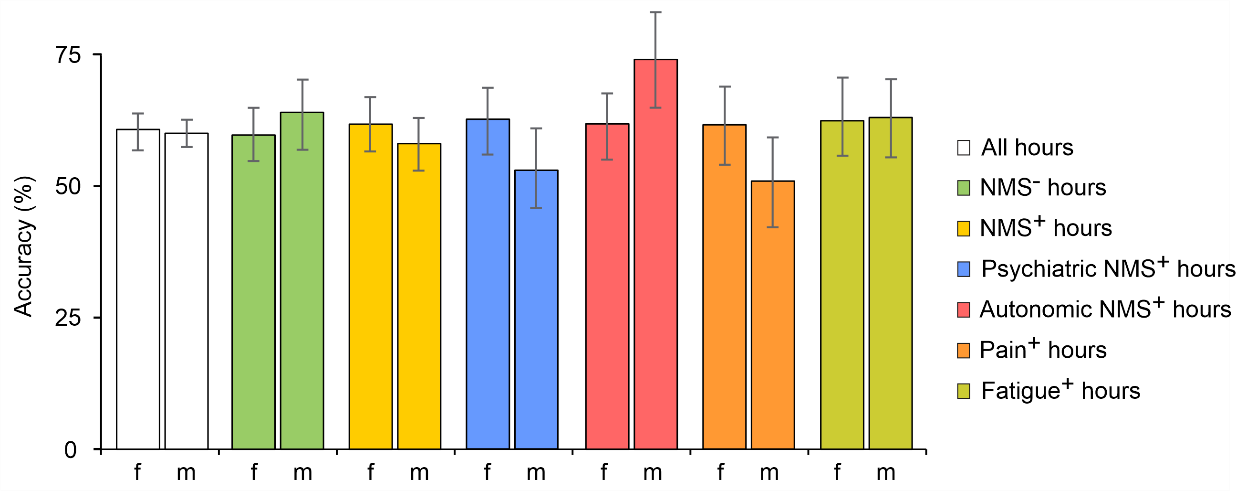


**Supplementary Figure S1 │ Overall accuracies of the PD Home motor diary when compared to observer ratings for the various NMS hours in dependency of sex.**

Diagram displays the overall accuracies of the PD Home diary in detecting the correct motor states reported by the professional observer PD diary with respect to sex (f=female; m=male). Values are presented as percentages with interquartile range (IQR) bars. IQR values were calculated using the Clopper-Pearson exact approximation. Accuracy values are reported for all hourly time periods, all hourly time periods with absence of NMS (NMS^-^), all hourly time periods with presence of NMS (NMS^+^), all hourly time periods with psychiatric NMS present (psychiatric NMS^+^), all hourly time periods with autonomic NMS present (autonomic NMS^+^), all hourly time periods with pain present (Pain^+^), and all hourly time periods with fatigue present (Fatigue^+^). We did not detect any differences of the accuracies between female and male participants for all comparisons with *P*≥0.05 (Pearson χ^2^ tests, unadjusted concerning α inflation).

**Supplementary Figure S2**


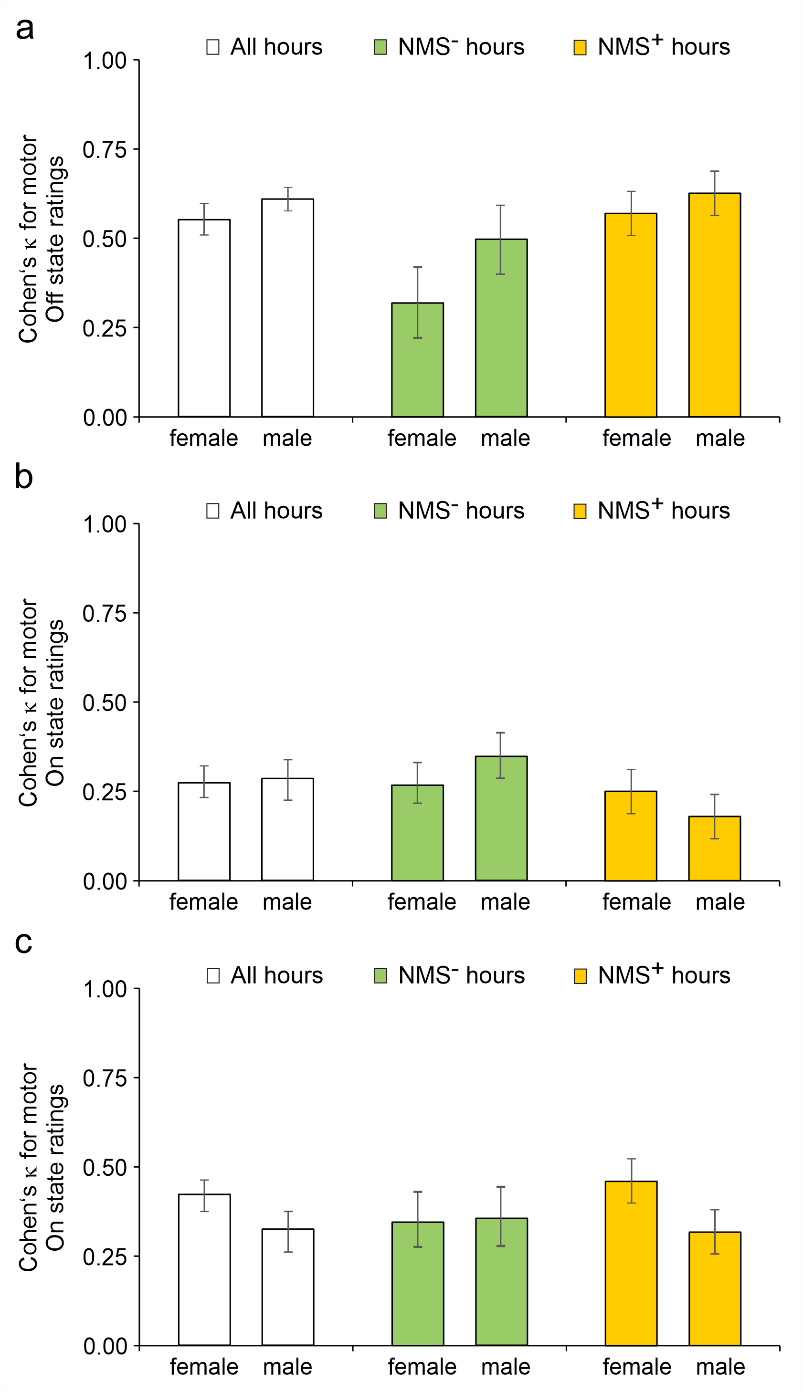


**Supplementary Figure S2 │ Cohen’s κ values of the PD Home motor diary when compared to observer ratings for the various NMS hours in dependency of sex.**

Bar charts comparing the performance measure Cohen’s κ during all hourly times (independent of NMS) as well as NMS^-^ and NMS^+^ hourly time periods with respect to sex of participants for **(a)** motor On state time periods, **(b)** motor Off state time periods and **(c)** Dyskinetic state time periods. Values are presented as κ values with IQR bars representing inter-rater agreement. IQR values were calculated using Clopper-Pearson exact approximation. We did not detect any differences of Cohen’s κ values between female and male participants for all comparisons *P*≥0.05 (Pearson χ^2^ tests, unadjusted concerning α inflation).

**Supplementary Figure S3**


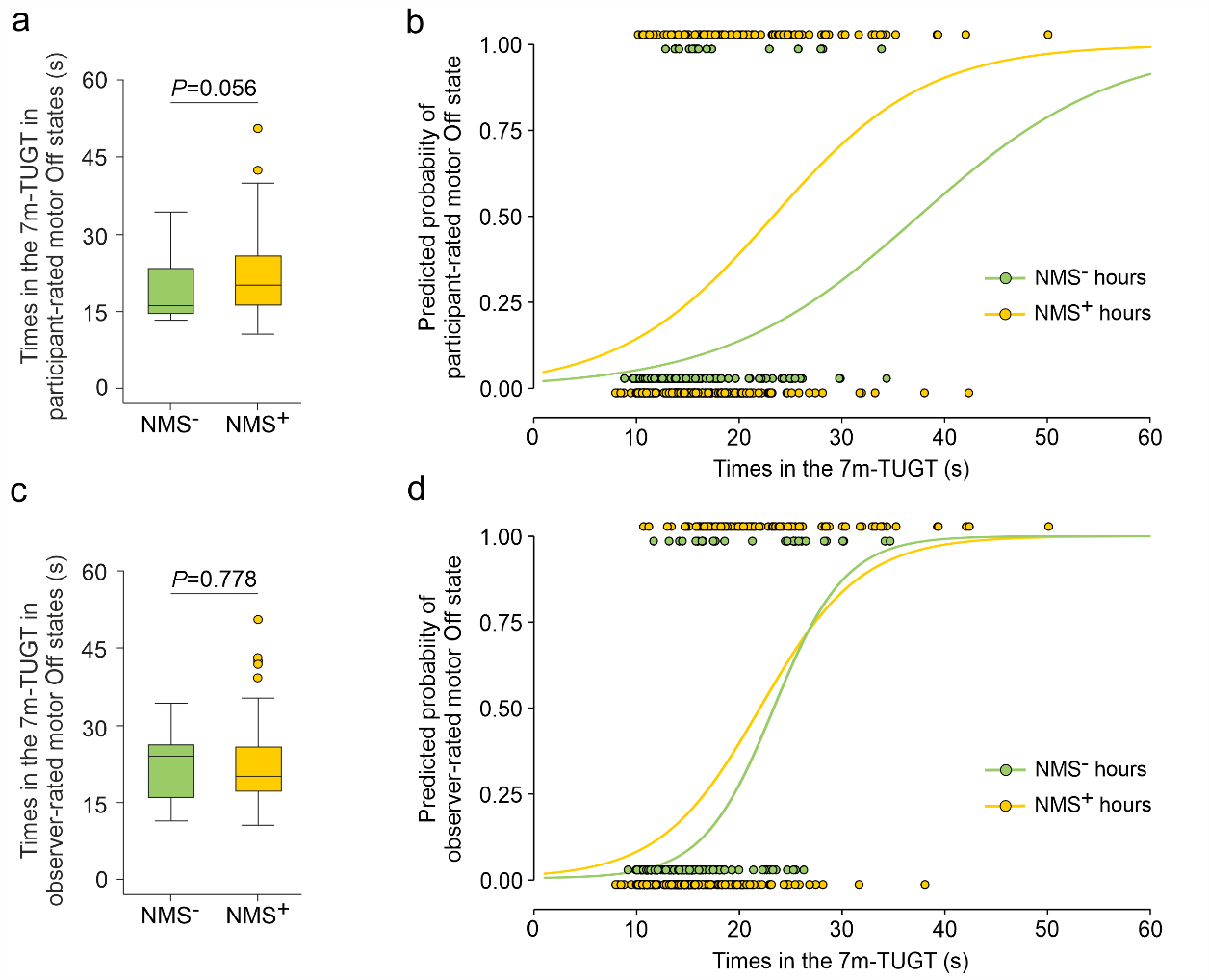


**Supplementary Figure S3 │ Relationship between times in the 7m-TUGT and diary Off ratings as documented by participants or observers.**

**(a,c)** Distribution of times in the 7m-TUGT expressed in seconds in motor Off states as rated by the participants in the PD Home diary **(a)** and by the clinical observer **(c)** with respect to simultaneous NMS occurrence. Boxplots are shown with a central mark at the median, bottom, and top edges of the boxes at 25^th^ and 75^th^ percentiles, respectively, whiskers out to the most extreme points within 1.5 times the interquartile range, and outliers scoring more than 1.5×IQR but at most 3×IQR outside the quartiles. Displayed *P*-values are from Mann-Whitney U-tests. **(b,d)** Fitted predicted probability plots from univariate logistic regression analyses. Times in the 7m-TUGT are plotted against the fitted predicted probability for motor Off ratings by the participants in the PD Home dairy **(b)** and by the clinical observer **(d)** with respect to NMS occurrence. For numeric results and statistical parameters of logistic regression analyses, please refer to ***Supplementary Table S8***.
