# Supplementary material for: Simultaneous Nonmotor Symptoms Do Not Affect General Validity but Interpretation of the Parkinson's Disease Motor Diary
Source: Mov Disord Clin Pract. 2025 Apr 3;12(7):979–84. doi: 10.1002/mdc3.70061 (PMC12275004; doi:10.1002/mdc3.70061)
Supplement: Supplementary file 2 — Table S1. Demographic and clinical characteristics of study cohort. Table S2. Diary data of waking day hours. Table S3. PD (Parkinson's disease) home motor diary test performance measures depending an NMS co‐occurence from 2 × 2 contingency tables for the detection of clinical observer ratings. Table S4. PD (Parkinson's disease) home motor diary test performance measures depending on NMS burden from 2 × 2 contingency tables for the detection of clinical observer ratings. Table S5. Validity parameters of the PD (Parkinson's disease) home diary for the detection of clinical observer Off ratings in balanced datasets. Table S6. Validity parameters of the PD (Parkinson's disease) home diary for the detection of clinical observer On ratings in balanced datasets. Table S7. Validity parameters of the PD (Parkinson's disease) home diary for the detection of observer dyskinetic state ratings in balanced datasets. Table S8. Effects of NMS (nonmotor symptom) occurrence and times in the 7 m‐TUGT (7‐meter Timed‐Up‐and‐Go‐Test) on likelihood of motor Off state ratings. [file MDC3-12-979-s001.docx]

**Supplementary Information – Tables**

**Simultaneous non-motor symptoms do not affect general validity but interpretation of the Parkinson’s Disease motor diary**

**Hampus Andersson, Alexander Bremer, Florin Gandor, Georg Ebersbach, Matthias Löhle, Per Odin, and Alexander Storch**

**Supplementary Tables:**

- **Supplementary Table S1.** Demographic and clinical characteristics of study cohort.
- **Supplementary Table S2.** Diary data of waking day hours.
- **Supplementary Table S3.** PD Home motor diary test performance measures from 2×2 contingency tables for the detection of clinical observer ratings.
- **Supplementary Table S4.** PD Home motor diary test performance measures from 2×2 contingency tables for the detection of clinical observer ratings.
- **Supplementary Table S5.** Validity parameters of the PD Home diary for the detection of clinical observer Off ratings in balanced datasets.
- **Supplementary Table S6.** Validity parameters of the PD Home diary for the detection of clinical observer On ratings in balanced datasets.
- **Supplementary Table S7.** Validity parameters of the PD Home diary for the detection of observer Dyskinetic state ratings in balanced datasets.
- **Supplementary Table S8.** Effects of NMS occurrence and times in the 7m-TUGT on likelihood of motor Off state ratings.

**Supplementary Table S1.** Demographic and clinical characteristics of study cohort.

|  | **Overall cohort (n=47)** |
| --- | --- |
| **Male / Female**, n (%) | 24 (51%) / 23 (49%) |
| **Age**, *Median (IQR) in years* | 65 (58-73) |
| **Disease duration***, Median (IQR) in years* | 10 (8-15) |
| **Symptom duration**, *Median (IQR) in years* | 12 (9-17) |
| **Duration of fluctuations**, *Median (IQR) in months* | 61 (34-106) |
| Hypokinetic fluctuations | 75 (40-114) |
| Hyperkinetic fluctuations | 38 (25-53) |
| **Type of motor complication**, *n (%)* |  |
| End-of dose akinesia | 44 (94%) |
| On-Off phenomenon | 31 (66%) |
| Delayed/no On response | 36 (77%) |
| Off period dystonia | 26 (55%) |
| Peak-dose dyskinesia | 36 (77%) |
| Biphasic dyskinesia | 9 (19%) |
| **Medication** |  |
| Total LED (mg per day), *Median (IQR)* | 1,325 (1,025-1,667) |
| **Clinical scales** |  |
| MDS-UPDRS Total score On state*,* *Median (IQR)* | 64 (52-83) |
| Part I | 13 (7-16) |
| Part II | 16 (10-20) |
| Part III | 28 (20-40) |
| Part IV | 9 (7-11) |
| Hoehn & Yahr stage, *Median (IQR)* | 2.5 (2-3) |
| Montreal Cognitive Assessment (MoCA) score, *Median (IQR)* | 27 (25-28) |
| Non-Motor Symptoms Scale (NMSS) score, *Median (IQR)* | 36 (29-60) |
| Beck’s Depression Inventory version 2 (BDI-2) score, *Median (IQR)* | 10 (4-16) |
| Parkinson’s Disease Questionnaire 39 (PDQ-39), *Median (IQR)* | 53 (33-67) |

Values are provided as number (percentages), median (interquartile range, IQR), or mean (standard deviation, SD). LED: Levodopa equivalent dose. MDS-UPDRS: Movement Disorder Society-revised version of the Unified Parkinson’s Disease Rating Scale. Levodopa equivalent doses were calculated in accordance with Jost and co-workers (Jost ST, Kaldenbach MA, Antonini A, et al. Levodopa dose equivalency in Parkinson's disease: Updated systematic review and proposals. Mov Disord 2023; 38: 1236-1252).

**Supplementary Table S2.** Diary data of waking day hours.

|  | **Number of hours (%)** |
| --- | --- |
| **PD Home motor diary (n=742 hours)** |  |
| Off state | 216 (29.1%) |
| On state | 368 (49.6%) |
| Dyskinetic state | 158 (21.3%) |
| **Clinical observer diary (n=503 hours)** |  |
| Off state | 145 (28.8%) |
| On state | 178 (35.4%) |
| Dyskinetic state | 180 (35.8%) |
| **NMS diary (n=688 hours)** |  |
| Total NMS | 423 (61.5%) |
| Psychiatric NMS | 222 (32.3%) |
| Autonomic NMS | 163 (23.7%) |
| Anxiety | 28 (4.1%) |
| Depressive mood | 75 (10.9%) |
| Fatigue | 198 (28.8%) |
| Inner restlessness | 165 (24.0%) |
| Concentration/attention | 92 (13.4%) |
| Hallucinations | 12 (1.7%) |
| Excessive sweating | 86 (12.5%) |
| Bladder urgency | 28 (4.1%) |
| Drooling | 39 (5.7%) |
| Dizziness | 57 (8.3%) |
| Pain | 191 (27.8%) |

Values are provided as number (percentages).

**Supplementary Table S3.** PD Home motor diary test performance measures from 2×2 contingency tables for the detection of clinical observer ratings.

|  | Accuracy (IQR) | | Balanced accuracy (IQR) | | | Sensitivity, recall (IQR) | | | Specificity (IQR) | | | PPV, precision (IQR) | | | NPV (IQR) | | | FPR (IQR) | | | FNR (IQR) | | | Cohen’s κ (IQR) | | MCC | | F1-score |
| --- | --- | --- | --- | --- | --- | --- | --- | --- | --- | --- | --- | --- | --- | --- | --- | --- | --- | --- | --- | --- | --- | --- | --- | --- | --- | --- | --- | --- |
| **Motor Off state hour time periods** | | | | |  | |  | | |  | | |  | | |  | | | |  |  | |  | |  | |  |  |
| All hour time periods | 84% (81-85%) | | 79% (75-82%) | | | 68% (63-73%) | | | 90% (88-92%) | | | 73% (68-77%) | | | 88% (85-90%) | | | 10% (8-12%) | | | 32% (27-37%) | | | 0.59 (0.54-0.63) | | 0.59 | | 0.70 |
| NMS^-^ hour time periods | 86% (83-89%) | | 69% (62-76%) | | | 42% (31-54%) | | | 95% (93-97%) | | | 65% (50-78%) | | | 89% (85-92%) | | | 5% (3-7%) | | | 58% (46-69%) | | | 0.43 (0.33-0.54) | | 0.45 | | 0.51 |
| NMS^+^ hour time periods | 82% (79-84%) | | 80% (76-84%) | | | 75% (70-80%)**^,$^ | | | 85% (82-88%)**^,$^ | | | 74% (69-79%) | | | 86% (83-89%) | | | 15% (12-18%)**^,$^ | | | 25% (20-30%)**^, $^ | | | 0.61 (0.55-0.66) | | 0.61 | | 0.75 |
| Psychiatric NMS present | 81% (77-85%) | | 82% (75-87%) | | | 84% (77-90%)^##,$^ | | | 80% (74-84%)^###,$^ | | | 69% (62-76%) | | | 90% (85-94%) | | | 20% (16-26%)^###, $^ | | | 16% (10-23%)^##, $^ | | | 0.61 (0.53-0.68) | | 0.61 | | 0.76 |
| Autonomic NMS present | 82% (78-86%) | | 81% (74-87%) | | | 75% (66-82%)^$^ | | | 87% (82-92%) | | | 80% (72-87%) | | | 83% (77-88%) | | | 13% (8-18%) | | | 25% (18-34%)^$^ | | | 0.63 (0.55-0.71) | | 0.63 | | 0.77 |
| Fatigue present | 82% (77-86%) | | 82% (75-87%) | | | 82% (74-88%)^#, $^ | | | 82% (76-87%)^##, $^ | | | 72% (64-79%) | | | 89% (84-93%) | | | 18% (13-24%)^##,$^ | | | 18% (12-26%)^#,$^ | | | 0.62 (0.54-0.70) | | 0.62 | | 0.77 |
| Pain present | 81% (76-85%) | | 79% (71-85%) | | | 74% (64-83%) | | | 83% (78- 88%)^#, $^ | | | 66% (56-75%) | | | 88% (83-92%) | | | 17% (12-22%)^#,$^ | | | 26% (17-36%) | | | 0.56 (0.47-0.65) | | 0.56 | | 0.70 |
| **Motor On state hour time periods** | |  | |  | | | |  | | |  | | |  | | |  | |  | | |  | |  | |  | |  |
| All hour time periods | 63% (61-66%) | | 65% (61-69%) | | | 71% (37-44%) | | | 60% (56-63%) | | | 49% (45-52%) | | | 79% (76-82%) | | | 40% (37-44%) | | | 29% (25-34%) | | | 0.27 (0.23-0.32) | | 0.29 | | 0.58 |
| NMS^-^ hour time periods | 62% (58-67%) | | 64% (58-69%) | | | 85% (79-89%) | | | 43% (37-49%) | | | 56% (51-62%) | | | 76% (68-83%) | | | 57% (51-63%) | | | 15% (11-21%) | | | 0.26 (0.19-0.34) | | 0.30 | | 0.68 |
| NMS^+^ hour time periods | 64% (61-68%) | | 62% (57-67%) | | | 58% (51-64%)***^,$^ | | | 67% (63-71%)***^,$^ | | | 41% (36-47%) | | | 80% (76-83%) | | | 33% (29-37%)***^,$^ | | | 42% (36-49%)***^,$^ | | | 0.22 (0.16-0.28) | | 0.23 | | 0.48 |
| Psychiatric NMS present | 64% (59-69%) | | 57% (49-64%) | | | 43% (33-53%)^###,$^ | | | 71% (66-76%)^##,$^ | | | 33% (25-42%) | | | 79% (73-83%) | | | 29% (24-34%)^##,$^ | | | 58% (47-67%)^###,$^ | | | 0.13 (0.03-0.22) | | 0.13 | | 0.37 |
| Autonomic NMS present | 69% (64-74%) | | 66% (57-74%) | | | 59% (48-70%) | | | 73% (67-78%)^##,$^ | | | 40% (32-50%) | | | 85% (80-89%) | | | 27% (22-33%)^##,$^ | | | 41% (30-52%) | | | 0.28 (0.18-0.37) | | 0.29 | | 0.48 |
| Fatigue present | 69% (64-74%) | | 63% (55-71%) | | | 50% (39-61%)^##,$^ | | | 75% (70-81%)^###,$^ | | | 42% (32-52%) | | | 81% (75-86%) | | | 25% (19-30%)^###,$^ | | | 50% (39-61%)^##,$^ | | | 0.24 (0.14-0.34) | | 0.24 | | 0.46 |
| Pain present | 64% (58-69%) | | 63% (54-70%) | | | 60% (49-69%)^$^ | | | 66% (59-72%)^$^ | | | 45% (37-54%) | | | 77% (70-83%) | | | 34% (28-41%)^$^ | | | 40% (31-51%)^$^ | | | 0.23 (0.13-0.33) | | 0.24 | | 0.52 |

*represents *P*<0.05, ** <0.01 and *** *P*<0.001 when compared to NMS^-^ hour time periods (Pearson χ^2^ test)

^#^represents *P*<0.05, ^##^ <0.01 and ^###^ *P*<0.001 when compared to NMS^-^ hour time periods for the respective NMS type (Pearson χ^2^ test)

^$^represents *P*<0.05 when compared to NMS^-^ hours using CI (estimated by Clopper-Pearson exact estimation)

**Supplementary Table S3 (cont.).** PD Home motor diary test performance measures from 2×2 contingency tables for the detection of clinical observer ratings.

|  | Accuracy (IQR) | Balanced accuracy (IQR) | | Sensitivity, recall (IQR) | | | Specificity (IQR) | PPV, precision (IQR) | | | NPV (IQR) | FPR (IQR) | | | FNR (IQR) | | Cohen’s κ (IQR) | | MCC | | F1-score | |
| --- | --- | --- | --- | --- | --- | --- | --- | --- | --- | --- | --- | --- | --- | --- | --- | --- | --- | --- | --- | --- | --- | --- |
| **Motor Dyskinetic state hour time periods** | | |  | |  |  | | |  |  | | |  |  | |  | |  | |  | |  |
| All hour time periods | 74% (71-76%) | 67% (64-70%) | | 44% (39-48%) | | | 90% (88-92%) | 71% (66-76%) | | | 74% (71-77%) | 10% (8-12%) | | | 56% (52-61%) | | 0.37 (0.32-0.42) | | 0.39 | | 0.54 | |
| NMS^-^ hour time periods | 73% (69-77%) | 65% (60-71%) | | 38% (31-46%) | | | 92% (88-95%) | 74% (63-83%) | | | 73% (68-77%) | 8% (5-12%) | | | 62% (54-69%) | | 0.34 (0.26-0.42) | | 0.38 | | 0.51 | |
| NMS^+^ hour time periods | 74% (71-77%) | 68% (63-72%) | | 46% (41-52%) | | | 89% (86-92%) | 71% (64-77%) | | | 75% (71-78%) | 11% (8-14%) | | | 54% (48-59%) | | 0.39 (0.32-45) | | 0.40 | | 0.56 | |
| Psychiatric NMS present | 72% (67-76%) | 67% (61-73%) | | 45% (37-53%) | | | 89% (84-92%) | 72% (62-80%) | | | 71% (66-76%) | 11% (8-16%) | | | 55% (47-63%) | | 0.36 (0.28-0.45) | | 0.38 | | 0.55 | |
| Autonomic NMS present | 81% (77-85%)^#^ | 77% (70-83%) | | 62% (52-70%)^##^ | | | 92% (87-95%) | 81% (70-88%) | | | 82% (76-86%) | 8% (5-13%) | | | 38% (30-48%)^##^ | | 0.57 (0.48-0.65) | | 0.58 | | 0.70 | |
| Fatigue present | 75% (71-77%) | 71% (64-77%) | | 54% (45-63%) | | | 88% (83-92%) | 74% (63-82%) | | | 76% (70-81%) | 12% (8-17%) | | | 46% (37-55%) | | 0.45 (0.36-0.54) | | 0.46 | | 0.62 | |
| Pain present | 69% (64-74%) | 63% (56-70%) | | 40% (31-49%) | | | 86% (81-91%) | 63% (51-74%) | | | 71% (65-76%) | 14% (9-19%) | | | 60% (51-69%) | | 0.28 (0.18-0.38) | | 0.30 | | 0.49 | |

*represents *P*<0.05, ** <0.01 and *** *P*<0.001 when compared to NMS^-^ hour time periods (Pearson χ^2^ test)

^#^represents *P*<0.05, ^##^ <0.01 and ^###^ *P*<0.001 when compared to NMS^-^ hour time periods for the respective NMS type (Pearson χ^2^ test)

^$^represents *P*<0.05 when compared to NMS^-^ hours using CI (estimated by Clopper-Pearson exact estimation)

**Supplementary Table S4.** PD Home motor diary test performance measures from 2×2 contingency tables for the detection of clinical observer ratings.

|  | Accuracy (IQR) | Balanced accuracy (IQR) | | | | | Sensitivity, recall (IQR) | | | | Specificity (IQR) | | | | PPV, precision (IQR) | | | | NPV (IQR) | | | FPR (IQR) | | | | FNR (IQR) | | | | | Cohen’s κ (IQR) | | | MCC | | | F1-Score | | | |
| --- | --- | --- | --- | --- | --- | --- | --- | --- | --- | --- | --- | --- | --- | --- | --- | --- | --- | --- | --- | --- | --- | --- | --- | --- | --- | --- | --- | --- | --- | --- | --- | --- | --- | --- | --- | --- | --- | --- | --- | --- |
| **Motor Off state hour time periods** | | |  | |  | | | | |  | | | |  | | | |  | | |  | |  | | | | | |  | | |  | | |  | | |  |  |  |
| NMS^-^ time periods | 86% (83-89%) | 69% (62-76%) | | | | | 42% (31-54%) | | | | 95% (93-97%) | | | | 65% (50-78%) | | | | 89% (85-92%) | | | 5% (3-7%) | | | | 58% (46-69%) | | | | | 0.43 (0.33-0.54) | | | 0.45 | | | 0.51 | | | |
| 1 NMS present | 85% (81-89%) | 81% (74-86%) | | | | | 67% (58-76%) | | | | 94% (90-96%) | | | | 84% (74-91%) | | | | 86% (81-90%) | | | 6% (4-10%) | | | | 32% (24-42%) | | | | | 0.64 (0.56-0.72) | | | 0.75 | | | 0.65 | | | |
| 2 NMS present | 82% (75-87%) | 81% (70-89%) | | | | | 78% (65-88%) | | | | 83% (74-90%) | | | | 72% (59-83%) | | | | 88% (79-93%) | | | 17% (10-26%) | | | | 22% (12-35%) | | | | | 0.60 (0.49-0.72) | | | 0.75 | | | 0.61 | | | |
| 3 NMS present | 68% (57-77%) | 68% (53-81%) | | | | | 76% (60-88%) | | | | 60% (45-74%)* | | | | 62% (47-75%) | | | | 75% (58-88%) | | | 40% (26-55%)* | | | | 24% (12-40%) | | | | | 0.36 (0.19-0.53) | | | 0.68 | | | 0.37 | | | |
| 4 NMS present | 84% (74-92%) | 85% (68-94%) | | | | | 83% (68-93%) | | | | 86% (68-96%) | | | | 88% (73-96%) | | | | 80% (62-92%) | | | 14% (4-32%) | | | | 17% (7-32%) | | | | | 0.69 (0.54-0.83) | | | 0.86 | | | 0.69 | | | |
| 5+ NMS present | 83% (72-90%) | 89% (70-100%) | | | | | 100% (74-100%) | | | | 78% (65-87%) | | | | 54% (35-72%) | | | | 100% (91-100%) | | | 22% (13-35%) | | | | 0% (0-26%) | | | | | 0.59 (0.43-0.75) | | | 0.70 | | | 0.65 | | | |
| **Motor On state hour time periods** | | |  |  | | | | |  | | | |  | | | |  | | | |  | | |  | | | |  | | | |  | | |  | | | |  |  |
| NMS^-^ time periods | 62% (58-67%) | 64% (58-69%) | | | | | 85% (79-89%) | | | | 43% (37-49%) | | | | 56% (51-62%) | | | | 76% (68-83%) | | | 57% (51-63%) | | | | 15% (11-21%) | | | | | 0.26 (0.19-0.34) | | | 0.30 | | | 0.68 | | | |
| 1 NMS present | 62% (57-67%) | 65% (57-72%) | | | | | 71% (62-79%) | | | | 58% (51-64%) | | | | 44% (37-52%) | | | | 81% (74-86%) | | | 42% (36-49%) | | | | 29% (21-38%) | | | | | 0.25 (0.16-0.34) | | | 0.55 | | | 0.37 | | | |
| 2 NMS present | 63% (55-70%) | 61% (48-72%) | | | | | 55% (25-43%) | | | | 67% (57-75%) | | | | 42% (30-55%) | | | | 77% (67-85%) | | | 33% (25-43%) | | | | 45% (31-60%) | | | | | 0.20 (0.06-0.34) | | | 0.48 | | | 0.20 | | | |
| 3 NMS present | 59% (49-70%) | 51% (35-68%) | | | | | 33% (14-58%)* | | | | 68% (55-79%) | | | | 25% (11-46%) | | | | 76% (63-86%) | | | 32% (21-45%) | | | | 67% (42-86%)* | | | | | 0.01 (-0.18-0.20) | | | 0,29 | | | 0.10 | | | |
| 4 NMS present | 75% (64-84%) | 69% (46-87%) | | | | | 60% (27-87%) | | | | 78% (65-87%)* | | | | 33% (14-58%) | | | | 91% (80-97%) | | | 22% (13-35%)* | | | | 40% (13-73%) | | | | | 0.28 (0.07-0.50) | | | 0.43 | | | 0.31 | | | |
| 5+ NMS present | 71% (59-80%) | 55% (42-71%)* | | | | | 22% (7-47%)* | | | | 88% (76-95%) | | | | 40% (13-73%) | | | | 76% (64-85%) | | | 12% (5-24%)* | | | | 78% (53-93%)* | | | | | 0.12 (-0.08-0.32) | | | 0.29 | | | 0.13 | | | |
| **Motor Dyskinetic state hour time periods** | | | | | |  | |  | | | |  | | | |  | | | |  | | | | |  | |  | | |  | | |  | | |  | | | |  |
| NMS^-^ time periods | 73% (69-77%) | 65% (60-71%) | | | | | 38% (31-46%) | | | | 92% (88-95%) | | | | 74% (63-83%) | | | | 73% (68-77%) | | | 8% (5-12%) | | | | 62% (54-69%) | | | | | 0.34 (0.26-0.42) | | | 0.39 | | | 0.54 | | | |
| 1 NMS present | 73% (68-77%) | 66% (59-72%) | | | | | 43% (34-52%) | | | | 89% (84-93%) | | | | 68% (56-78%) | | | | 74% (69-79%) | | | 11% (7-16%) | | | | 57% (48-66%) | | | | | 0.35 (0.25-0.44) | | | 0.53 | | | 0.37 | | | |
| 2 NMS present | 72% (65-79%) | 65% (54-75%) | | | | | 41% (28-55%) | | | | 88% (80-94%) | | | | 64% (45-80%) | | | | 75% (66-82%) | | | 12% (6-20%) | | | | 59% (45-72%) | | | | | 0.32 (0.18-0.46) | | | 0.50 | | | 0.34 | | | |
| 3 NMS present | 76% (65-84%) | 65% (51-77%) | | | | | 33% (17-54%) | | | | 96% (86-99%) | | | | 80% (44-97%) | | | | 75% (64-84%) | | | 4% (0.5-14%) | | | | 67% (46-83%) | | | | | 0.35 (0.17-0.53) | | | 0.47 | | | 0.40 | | | |
| 4 NMS present | 84% (74-92%) | 76% (59-88%) | | | | | 56% (32-77%) | | | | 96% (85-99%) | | | | 83% (52-98%) | | | | 85% (73-93%) | | | 4% (0.6-15%) | | | | 44% (23-68%) | | | | | 0.57 (0.38-0.76) | | | 0.67 | | | 0.59 | | | |
| 5+ NMS present | 71% (59-80%) | 71% (54-84%) | | | | | 67% (50-80%) | | | | 75% (58-88%) | | | | 75% (58-88%) | | | | 67% (50-80%) | | | 25% (12-42%) | | | | 33% (20-50%) | | | | | 0.41 (0.24-0.59) | | | 0.71 | | | 0.42 | | | |

*represents *P*<0.05, ** *P*<0.01 and *** *P*<0.001 when compared to NMS^-^ hour time periods (Pearson χ^2^ test)

**Supplementary Table S5.** Validity parameters of the PD Home diary for the detection of clinical observer Off ratings in balanced datasets.

|  | Accuracy (IQR) | Balanced accuracy (IQR) | Sensitivity, recall (IQR) | Specificity (IQR) | PPV, precision (IQR) | NPV (IQR) | FPR (IQR) | FNR (IQR) | Cohen’s κ (IQR) | MCC | F1-score | |
| --- | --- | --- | --- | --- | --- | --- | --- | --- | --- | --- | --- | --- |
| **All hour Off state time periods** | | | | | | | | |  |  |  |  |
| Original dataset | 84% (81-85%) | 79% (75-82%) | 68% (63-73%) | 90% (88-92%) | 73% (68-77%) | 88% (85-90%) | 10% (8-12%) | 32% (27-37%) | 0.59 (0.54-0.63) | 0.59 | 0.70 |  |
| Randomly undersampled balanced dataset | 83% (80-85%) | 83% (79-86%) | 68% (63-72%) | 98% (96-99%) | 97% (94-99%)** | 75% (71-79%) | 2% (1-4%)* | 32% (28-37%) | 0.66 (0.61-0.70) | 0.69 | 0.79 | |
| Randomly oversampled balanced dataset | 79% (77-81%) | 79% (76-81%) | 68% (65-71%) | 90% (88-92%) | 87% (84-89%)* | 74% (71-76%)* | 10% (8-12) | 32% (29-35%) | 0.58 (0.54-0.64) | 0.60 | 0.76 | |
| Randomly combined under-/oversampled dataset | 87% (85-88%) | 87% (84-89%) | 81% (78-84%)* | 92% (90-94%) | 91% (88-93%)** | 83% (80-86%) | 8% (6-10%) | 19% (16-22%)* | 0.73 (0.70-0.77) | 0.74 | 0.86 | |
| **NMS^-^ hour Off state time periods** | | | | | | | | |  |  |  |  |
| Original dataset | 86% (83-89%) | 69% (62-76%) | 42% (31-54%) | 95% (93-97%) | 65% (50-78%) | 89% (85-92%) | 5% (3-7%) | 58% (46-69%) | 0.43 (0.33-0.54) | 0.45 | 0.51 | |
| Randomly undersampled balanced dataset | 66% (58-73%)* | 66% (56-75%) | 42% (31-54%) | 90% (80-96%) | 81% (64-92%) | 61% (51-70%)** | 9% (4-19%) | 58% (46-69%) | 0.32 (0.19-0.46) | 0.37 | 0.55 | |
| Randomly oversampled balanced dataset | 72% (66-79%) | 72% (66-80%) | 40% (29-51%) | 89% (79-91%) | 76% (62-86%) | 63% (55-74%)* | 7% (3-15%) | 57% (44-67%) | 0.35 (0.21-0.48) | 0.38 | 0.53 | |
| Randomly combined under-/oversampled dataset | 82% (78-85%) | 82% (78-86%) | 41% (39-45%) | 93% (83-97%) | 78% (65-92%) | 75% (61-82%) | 6% (3-11%) | 57% (45-68%) | 0.42 (0.31-0.52) | 0.39 | 0.52 | |
| **NMS^+^ hour Off state time periods** | | | | | | | | |  |  |  |  |
| Original dataset | 82% (79-84%) | 80% (76-84%) | 75% (70-80%) | 85% (82-88%) | 74% (69-79%) | 86% (83-89%) | 15% (12-18%) | 25% (20-30%) | 0.61 (0.55-0.66) | 0.61 | 0.75 | |
| Randomly undersampled balanced dataset | 81% (78-84%) | 81% (76-86%) | 75% (70-80%) | 87% (83-91%) | 86% (80-90%) | 78% (73-82%) | 13% (9-17%) | 25% (20-30%) | 0.63 (0.57-0.69) | 0.63 | 0.80 | |
| Randomly oversampled balanced dataset | 80% (77-85%) | 80% (76-87%) | 72% (68-77%) | 83% (80-89%) | 88% (83-94%)* | 72% (69-76%)* | 5% (3-9%)* | 25% (219-30%) | 0.55 (0.52-0.67) | 0.61 | 0.79 | |
| Randomly combined under-/oversampled dataset | 82% (78-84%) | 82% (75-87%) | 74% (70-81%) | 86% (82-92%) | 85% (82-91%)* | 77% (72-81%) | 11% (7-13%) | 25% (21-30%) | 0.73 (0.68-0.78) | 0.59 | 0.77 | |

*represents *P*<0.05, ** *P*<0.01 and *** *P*<0.001 when compared to original dataset (Pearson χ^2^ test)

**Supplementary Table S6.** Validity parameters of the PD Home diary for the detection of clinical observer On ratings in balanced datasets.

|  | Accuracy (IQR) | Balanced accuracy (IQR) | Sensitivity, recall (IQR) | Specificity (IQR) | PPV, precision (IQR) | NPV (IQR) | FPR (IQR) | FNR (IQR) | Cohen’s κ (IQR) | MCC | F1-score | |
| --- | --- | --- | --- | --- | --- | --- | --- | --- | --- | --- | --- | --- |
| **All hour On state time periods** | | | | | | | | |  |  |  |  |
| Original dataset | 63% (61-66%) | 65% (61-69%) | 71% (67-74%) | 60% (56-63%) | 49% (45-52%) | 79% (76-82%) | 40% (37-44%) | 29% (25-34%) | 0.27 (0.23-0.32) | 0.29 | 0.58 |  |
| Randomly undersampled balanced dataset | 58% (55-61%) | 58% (54-62%) | 70% (66-74%) | 46% (42-51%) | 56% (52-61%) | 61% (55-66%) | 54% (49-58%) | 30% (26-34%) | 0.16 (0.10-0.22) | 0.17 | 0.63 | |
| Randomly oversampled balanced dataset | 68% (66-70%) | 68% (65-71%) | 76% (73-79%) | 60% (56-63%) | 65% (62-68%)* | 71% (68-75%) | 40% (37-44%) | 24% (21-27%) | 0.36 (0.32-0.40) | 0.36 | 0.70 | |
| Randomly combined under-/oversampled dataset | 74% (71-76%)* | 74% (70-77%) | 84% (81-87%)* | 63% (60-67%) | 70% (66-73)** | 80% (76-83%) | 36% (32-40%) | 16% (13-19%)* | 0.38 (0.32-0.42) | 0.39 | 0.66 | |
| **NMS^-^ hour On state time periods** | | | | | | | | |  |  |  |  |
| Original dataset | 62% (58-67%) | 64% (58-69%) | 85% (79-89%) | 43% (37-49%) | 56% (51-62%) | 76% (68-83%) | 57% (51-63%) | 15% (11-21%) | 0.26 (0.19-0.34) | 0.30 | 0.68 | |
| Randomly undersampled balanced dataset | 67% (62-71%) | 67% (60-72%) | 84% (79-89%) | 49% (42-56%) | 62% (57-68%) | 76% (68-83%) | 51% (44-58%) | 15% (11-21%) | 0.33 (0.26-0.41) | 0.36 | 0.72 | |
| Randomly oversampled balanced dataset | 66% (61-70%) | 66% (60-72%) | 83% (78-89%) | 48% (41-56%) | 64% (59-70%) | 77% (69-85%) | 41% (35-49%) | 14% (11-21%) | 0.38 (0.27-0.47) | 0.32 | 0.71 | |
| Randomly combined under-/oversampled dataset | 64% (61-70%) | 64% (60-71%) | 85% (79-90%) | 50% (45-58%)* | 63% (58-69%) | 76% (69-82%) | 51% (43-58%) | 13% (10-20%) | 0.37 (0.31-0.41) | 0.37 | 0.66 | |
| **NMS^+^ hour On state time periods** | | | | | | | | |  |  |  |  |
| Original dataset | 64% (61-68%) | 62% (57-67%) | 58% (51-64%) | 67% (63-71%) | 41% (36-47%) | 80% (76-83%) | 33% (29-37%) | 42% (36-49%) | 0.22 (0.16-0.28) | 0.23 | 0.48 | |
| Randomly undersampled balanced dataset | 60% (55-66%) | 60% (54-67%) | 58% (51-64%) | 63% (56-69%) | 61% (54-67%)* | 60% (53-66%)* | 38% (31-44%) | 42% (36-49%) | 0.20 (0.14-0.27) | 0.20 | 0.59 | |
| Randomly oversampled balanced dataset | 62% (53-69%) | 62% (53-70%) | 60% (53-68%) | 65% (58-71%) | 64% (56-69%)* | 66% (59-75%) | 33% (28-43%) | 36% (31-45%) | 0.24 (0.17-0.32) | 0.25 | 0.51 | |
| Randomly combined under-/oversampled dataset | 63% (58-67%) | 63% (58-69%) | 59% (51-64%) | 66% (62-70%) | 64% (56-69%)* | 69% (61-77%)* | 33% (30-42%) | 21% (15-31%)* | 0.21 (0.15-0.27) | 0.21 | 0.50 | |

*represents *P*<0.05, ** *P*<0.01 and *** *P*<0.001 when compared to original dataset (Pearson χ^2^ test)

**Supplementary Table S7.** Validity parameters of the PD Home diary for the detection of observer Dyskinetic state ratings in balanced datasets.

|  | Accuracy (IQR) | Balanced accuracy (IQR) | Sensitivity, recall (IQR) | Specificity (IQR) | PPV, precision (IQR) | NPV (IQR) | FPR (IQR) | FNR (IQR) | Cohen’s κ (IQR) | MCC | F1-score | |
| --- | --- | --- | --- | --- | --- | --- | --- | --- | --- | --- | --- | --- |
| **All hour Dyskinetic state time periods** | | | | | | | | |  |  |  |  |
| Original dataset | 74% (71-76%) | 67% (64-70%) | 44% (39-48%) | 90% (88-92%) | 71% (66-76%) | 74% (71-77%) | 10% (8-12%) | 56% (52-61%) | 0.37 (0.32-0.42) | 0.39 | 0.54 |  |
| Randomly undersampled balanced dataset | 69% (66-72%) | 69% (65-72%) | 44% (39-49%) | 94% (91-96%) | 88% (83-92%)* | 63% (59-66%)* | 1% (0-2%)** | 56% (52-61%) | 0.38 (0.33-0.42) | 0.44 | 0.58 | |
| Randomly oversampled balanced dataset | 73% (71-75%) | 73% (70-76%) | 56% (52-59%)* | 90% (88-92%) | 85% (82-88%)* | 67% (64-70%) | 10% (7-12%) | 44% (41-48%)* | 0.46 (0.42-0.50) | 0.49 | 0.67 | |
| Randomly combined under-/oversampled dataset | 69% (67-72%) | 69% (67-73%) | 48% (45-52%) | 91% (89-93%) | 84% (80-88%)* | 64% (61-67%)* | 9% (7-11%)* | 52% (48-55%) | 0.40 (0.35-0.44) | 0.44 | 0.62 | |
| **NMS^-^ hour Dyskinetic state time periods** | | | | | | | | |  |  |  |  |
| Original dataset | 73% (69-77%) | 65% (60-71%) | 38% (31-45%) | 92% (88-95%) | 74% (63-83%) | 73% (68-77%) | 8% (5-12%) | 62% (54-69%) | 0.34 (0.26-0.42) | 0.38 | 0.51 | |
| Randomly undersampled balanced dataset | 63% (57-67%) | 63% (55-69%) | 38% (31-46%) | 86% (80-91%) | 74% (63-83%) | 58% (52-64%)* | 14% (9-20%) | 62% (54-69%) | 0.25 (0.15-0.34) | 0.28 | 0.51 | |
| Randomly oversampled balanced dataset | 65% (59-71%) | 65% (59-72%) | 51% (49-61%)* | 81% (74-87%) | 75% (64-85%) | 55% (49-62%)* | 4% (1-7%) | 51% (47-56%) | 0.45 (0.35-0.54) | 0.41 | 0.50 | |
| Randomly combined under-/oversampled dataset | 69% (61-73%) | 69% (61-74%) | 41% (34-49%) | 86% (81-91%) | 78% (68-89%) | 57% (50-62%)* | 9% (3-12%) | 62% (55-68%) | 0.35 (0.25-0.44) | 0.38 | 0.56 | |
| **NMS^+^ hour Dyskinetic state time periods** | | | | | | | | |  |  |  |  |
| Original dataset | 74% (71-77%) | 68% (63-72%) | 46% (41-52%) | 89% (86-92%) | 71% (64-77%) | 75% (71-78%) | 11% (8-14%) | 54% (48-59%) | 0.39 (0.32-45) | 0.40 | 0.56 | |
| Randomly undersampled balanced dataset | 69% (65-73%) | 69% (64-74%) | 46% (41-52%) | 92% (88-95%) | 85% (78-90%) | 63% (58-68%) | 8% (5-12%) | 54% (48-59%) | 0.38 (0.32-0.45) | 0.43 | 0.60 | |
| Randomly oversampled balanced dataset | 69% (64-74%) | 69% (64-76%) | 59% (51-64%) | 88% (82-95%) | 84% (76-91%)* | 60% (54-62%)* | 5% (2-9%) | 43% (39-49%) | 0.48 (0.42-0.55) | 0.45 | 0.61 | |
| Randomly combined under-/oversampled dataset | 68% (66-73%) | 68% (66-75%) | 47% (41-54%) | 91% (87-95%) | 79% (73-84%) | 70% (62-74%) | 9% (5-13%) | 53% (47-59%) | 0.41 (0.35-0.49) | 0.43 | 0.59 | |

*represents *P*<0.05, ** *P*<0.01 and *** *P*<0.001 when compared to original dataset (Pearson χ^2^ test)

**Supplementary Table S8.** Effects of NMS occurrence and times in the 7m-TUGT on likelihood of motor Off state ratings.

|  | NMS occurrence^§^ |  | | Times in 7m-TUGT^#^ | | | | | | | |  | NMS occurrence and 7m-TUGT^$^ | | | | | | | | | | | |
| --- | --- | --- | --- | --- | --- | --- | --- | --- | --- | --- | --- | --- | --- | --- | --- | --- | --- | --- | --- | --- | --- | --- | --- | --- |
|  | Odds ratio (95% CI) |  | | Odds ratio (95%CI) | | | χ^2^ (*P*-value*)*; Nagelkerke R^2^; correctly classified ratings | | | | |  | NMS: Odds ratio (95%CI) | | | | 7m-TUGT: Odds ratio (95%CI) | | | | χ^2^ (*P*-value*)*; Nagelkerke R^2^; correctly classified ratings | | | |
| **Motor Off state ratings by participants using the PD Home diary** | | | | | | | |  |  | |  | | | | | | |  |  | | |  |  | |
| NMS^-^ hour time periods | - |  | | **1.11 (1.02-1.206)*** | | | χ^2^: 5.9 (*P*=0.015); R^2^: 7%; correct ratings: 90% | | | | |  | - | | | | - | | | | - | | | |
| NMS^+^ hour time periods | **3.68 (2.46-5.51)***** |  | | **1.14 (1.09-1.20)***** | | | χ^2^: 42.0 (*P*<0.001); R^2^: 19%; correct ratings: 74% | | | | |  | **3.80 (2.15-6.70)***** | | | | **1.14 (1.09-1.18)***** | | | | χ^2^: 84.8 (*P<*0.001); R^2^: 25%; correct ratings: 79% | | | |
| Psychiatric NMS present | **2.74 (1.93-3.87)***** |  | | **1.10 (1.04-1.27)***** | | | χ^2^: 13.8 (*P*<0.001); R^2^: 13%; correct ratings: 66% | | | | |  | **2.86 (1.79-4.56)***** | | | | **1.14 (1.09-1.18)***** | | | | χ^2^: 79.4 (*P<*0.001); R^2^: 23%; correct ratings: 78% | | | |
| Autonomic NMS present | **1.64 (1.12-2.38)*** |  | | **1.12 (1.05-1.19)***** | | | χ^2^: 15.1 (*P*<0.001); R^2^: 16%; correct ratings: 71% | | | | |  | **1.70 (1.05-2.76)*** | | | | **1.14 (1.10-1.18)***** | | | | χ^2^: 65.0 (*P<*0.001); R^2^: 19%; correct ratings: 79% | | | |
| Fatigue present | **2.42 (1.70-3.44)***** |  | | **1.08 (1.03-1.14)**** | | | χ^2^: 9.7 (*P*=0.002); R^2^: 16%; correct ratings: 65% | | | | |  | **1.96 (1.21-3.18)**** | | | | **1.14 (1.10-1.18)***** | | | | χ^2^: 67.7 (*P<*0.001); R^2^: 20%; correct ratings: 77% | | | |
| Pain present | **1.63 (1.14-2.34)**** |  | | **1.09 (1.03-1.16)**** | | | χ^2^: 9.2 (*P*=0.002); R^2^: 16%; correct ratings: 69% | | | | |  | 1.33 (0.81-2.18) | | | | **1.14 (1.10-1.19)***** | | | | χ^2^: 61.7 (*P<*0.001); R^2^: 18%; correct ratings: 77% | | | |
| **Motor Off state ratings by clinical observer** | | |  | |  |  | | | |  | | | |  |  |  | | | |  | | | |  |
| NMS^-^ hour time periods | - |  | | **1.33 (1.208-1.47)***** | | | χ^2^: 47.2 (*P*<0.001); R^2^: 40%; correct ratings: 90% | | | | |  | - | | | | - | | | | - | | | |
| NMS^+^ hour time periods | 1.69 (0.99-4.21) |  | | **1.23 (1.16-1.30)***** | | | χ^2^: 77.0 (*P*<0.001); R^2^: 33%; correct ratings: 74% | | | | |  | 1.94 (0.94-3.39) | | | | **1.26 (1.20-1.32)***** | | | | χ^2^: 140.1 (*P<*0.001); R^2^: 38%; correct ratings: 80% | | | |
| Psychiatric NMS present | **1.57 (1.05-2.37)*** |  | | **1.18 (1.10-1.26)***** | | | χ^2^: 33.0 (*P*<0.001); R^2^: 28%; correct ratings: 73% | | | | |  | 1.01 (0.60-1.70) | | | | **1.27 (1.20-1.33)***** | | | | χ^2^: 134.2 (*P<*0.001); R^2^: 37%; correct ratings: 80% | | | |
| Autonomic NMS present | 1.12 (0.78-3.36) |  | | **1.15 (1.07-1.23)***** | | | χ^2^: 21.8 (*P*<0.001); R^2^: 21%; correct ratings: 68% | | | | |  | 1.62 (0.96-2.74) | | | | **1.26 (1.19-1.32)***** | | | | χ^2^: 137.8 (*P<*0.001); R^2^: 37%; correct ratings: 81% | | | |
| Fatigue present | **1.66 (1.09-2.53)*** |  | | **1.15 (1.08-1.22)***** | | | χ^2^: 24.0 (*P*<0.001); R^2^: 24%; correct ratings: 73% | | | | |  | 0.79 (0.45-1.38) | | | | **1.27 (1.21-1.34)***** | | | | χ^2^: 135.2 (*P<*0.001); R^2^: 37%; correct ratings: 79% | | | |
| Pain present | 1.10 (0.71-1.70) |  | | **1.14 (1.07-1.23)***** | | | χ^2^: 19.0 (*P*<0.001); R^2^: 20%; correct ratings: 76% | | | | |  | 0.58 (0.33-1.03) | | | | **1.28 (1.21-1.34)***** | | | | χ^2^: 138.1 (*P<*0.001); R^2^: 37%; correct ratings: 79% | | | |

^§^data from Pearson χ^2^ tests with respective NMS^-^ hour time periods as reference. *represents *P*<0.05, ** *P*<0.01 and *** *P*<0.001 (unadjusted concerning α inflation)

^#^data from univariate binary logistic regressions with times in 7m-TUGT as independent variable. *represents *P*<0.05, ** *P*<0.01 and *** *P*<0.001 (unadjusted concerning α inflation)

^$^data from multivariate binary logistic regressions with respective NMS occurrence and times in 7m-TUGT as independent variables (respective NMS^-^ time periods served as reference). *represents *P*<0.05, ** *P*<0.01 and *** *P*<0.001 (unadjusted concerning α inflation)
