# Supplementary material for: Simultaneous Nonmotor Symptoms Do Not Affect General Validity but Interpretation of the Parkinson's Disease Motor Diary
Source: Mov Disord Clin Pract. 2025 Apr 3;12(7):979–84. doi: 10.1002/mdc3.70061 (PMC12275004; doi:10.1002/mdc3.70061)
Supplement: Supplementary file 3 — Data S1. Supporting information. [file MDC3-12-979-s003.docx]

**Supplementary Information – Text**

**Simultaneous non-motor symptoms do not affect general validity but interpretation of the Parkinson’s Disease motor diary**

**Hampus Andersson, Alexander Bremer, Florin Gandor, Georg Ebersbach, Matthias Löhle, Per Odin, and Alexander Storch**

**Supplementary Text:**

- **Supplementary Methods.** Study synopsis.
- **Supplementary Methods.** Study participants.
- **Supplementary Methods.** Baseline assessments.
- **Supplementary Methods.** Statistical methods.
- **Supplementary Methods.** Balancing datasets.
- **Supplementary Results.** Demographic and clinical characteristics of study cohort.
- **Supplementary Results.** Diary adherence.
- **Supplementary Results.** Diary data on motor and non-motor symptom patterns.
- **Supplementary Results.** Data imbalance and PD Home diary test performance/validity measures.
- **Supplementary Results.** Influence of sex on PD Home diary test performance/validity measures.
- **Supplementary Results.** Times in the 7-meter Timed-Up-and-Go-Test and NMS diary ratings.
- **Supplementary Results.** Relationship between 7m-TUGT results and PD Home diary motor Off state ratings
- **Supplementary Results.** Relationship between 7m-TUGT results and observer-documented motor Off state ratings.
- **Supplementary Discussion.** Study limitations.

**SUPPLEMENTARY METHODS**

**Study synopsis.**

The German part of the VALIDATE PD study was conducted at two hospital centers (University Medicine Rostock, Movement Disorder Clinic Beelitz-Heilstätten) between October 2017 and July 2019.^1,2^ The study was approved by the institutional review boards of both participating centers (registry numbers A 2017-0115 for Rostock and AS 84(bB)/2018 for Beelitz-Heilstätten). All participants provided written informed consent before study participation.

After detailed instructions on the PD home diary and watching a training video explaining all functional states with particular focus on the difference between tremor and dyskinesia,^3^ the initial day (day 0) was used for diary training and to ensure sufficient adherence to the motor diary. Participants were visited by their treating neurologists, who checked diaries for inconsistencies and addressed potential questions. Data from day 0 were not used for outcome analyses. On the following two days (days 1 and 2), participants were asked to indicate their predominant status during hour time periods for two consecutive days using the categories Asleep, Off, On without dyskinesia, On with non-troublesome dyskinesia and On with troublesome dyskinesia (herein combined as Dyskinetic state).

On study days 1 and 2, participants were simultaneously observed by an experienced physiotherapist (A.B.), who had been trained to identify motor complications in advanced PD patients in the participating hospitals and acquired MDS certification as qualified UPDRS rater prior to the start of the study. The observer acted as single rater in our study and independently evaluated motor states throughout daytime (8.00 am through 6.00 pm) based on his clinical observations during active motor performance over the course of the 7-meter version of the Timed-Up-and-Go-Test (7m-TUGT),^4^ taking into account global bradykinesia, tremor, dyskinesia and gait function. All 7m-TUGT times were recorded but not considered for the observer ratings. The observer was also instructed to dismiss any attempts from patients to get assistance with their own ratings.

On study day 1, participants were asked to rate eleven key NMS (psychiatric NMS: anxiety, depressive mood, inner restlessness, difficulties with concentration, hallucinations; autonomic NMS: excessive sweating, sialorrhea, bladder urgency and dizziness; fatigue and pain) as present or absent during awake time on an hourly basis using the same questions as the NMSQuest as already introduced by Ossig and colleagues.^5^ NMS burden was herein defined as the number of co-occurring NMS. Motor diaries were originally completed on a half-hourly basis, but here the motor data was used, which was collected at the same time as the NMS data. On day 0 prior to conducting NMS diary assessments, participants were trained in the definitions of the various NMS states. Data from day 1 were used for outcome analyses in the present report. On study day 2, participants were asked to rate pain using a pain diary. These data are published by Storch and colleagues^6^ and not used in the present analyses.

**Study participants.**

Study participants were selected based on the following inclusion and exclusion criteria: All selected participants had to be over the age of 30 years. Their PD diagnosis had to be based on the United Kingdom PD Society Brain Bank criteria. Furthermore, all participants had to experience motor fluctuations, verified either by their respective treating physicians or by part IV of the Movement Disorder Society-revised Unified Parkinson’s Disease Rating Scale (MDS-UDPRS).

Potential study participants showing signs of presence of dementia, as assessed by the Montreal Cognitive Assessment (MoCA score <21),^7^ were excluded from further participation in the study. Further exclusion criteria comprised the existence of any clinical signs for secondary or atypical parkinsonian syndromes, inability to complete questionnaires and/or patient diaries, lack of cooperation during the study procedures and/or relevant psychotic symptoms, ongoing treatment with advanced/invasive therapies (deep brain stimulation, subcutaneous apomorphine and levodopa-carbidopa intestinal gel) as well as the presence of miscellaneous diseases impairing the ability for consenting, participation and judgment in the patient.

**Baseline assessments.**

Baseline assessment include demographic and clinical data including PD medication, clinical phenotype, type of motor complication, Hoehn-Yahr score,^8^ MDS-UPDRS,^9^ cognitive screening with Montreal Cognitive Assessment (MoCA),^7^ the Non-Motor Symptom Scale (NMSS),^10^ Beck’s Depression Inventory version 2 (BDI-II),^11^ and the PD Questionnaire (PDQ-39) to assess health-related quality of life.^12^ The levodopa equivalent dose (LED) was calculated according to the method developed by Jost and co-workers.^13^

**Statistical methods.**

Cohen’s κ values were interpreted using the guidelines established by Cicchetti in 1994:^14^ Cohen’s κ values of κ<0.40 were considered poor, κ=0.40-0.59 were interpreted as moderate, κ=0.60-0.74 were deemed good and κ=0.75-1.00 were considered to represent excellent inter-rater agreement. The Pearson χ^2^ test was used for statistical comparisons of accuracy, sensitivity, specificity, false positive rate (FPR) and false negative rate (FNR). Determination of the 95% confidence interval (95%CI) and IQR for the performance measures (except for Cohen’s κ) was performed using the Clopper-Pearson exact method. Standard estimation using Wald approximation yielded almost identical results. Values provided by the Clopper-Pearson exact approximation were chosen as this statistical analysis is more conservative for small data sets.^15^ The 95%CI of Cohen’s κ values was estimated using its standard error.

Binary logistic regression was performed to ascertain the effects of times in the 7m-TUGT and NMS diary ratings on the likelihood of motor Off state ratings in simultaneous PD Home or observer-documented diary assessments using the SPSS software version 28 (IBM Cooperation, New York, USA). Assumptions were addressed for linearity of 7m-TGUT data with the logit transformed motor Off ratings using the Box-Tidwell approach, independency of observations, excluding outliers and the absence of multicollinearity. As NMS diary ratings, we tested total NMS occurrence, and co-occurring aggregated psychiatric NMS and autonomic NMS as well as pain and fatigue.

Handling of missing or inconclusive data was performed as follows: Individual time periods faced exclusion when response was missing, there were more than one response on either the motor or NMS diary, or if the participant had marked Asleep in one diary and not the other.

**Balancing datasets.**

Imbalance of outcome classes was estimated as the imbalance ratio (IR) defined as the number of majority class samples (negative ratings) divided by the number of minority class samples (positive ratings) and values of 5 or higher were considered as relevant (moderate to severe) imbalance.^16^ Although diary outcome data were not or only mildly imbalanced with IR values between 1.14 and 4.76 for clinical observer diary data as the outside validation criterion,^16^ we addressed the challenge of class imbalance by balancing the data using random undersampling the majority class, random oversampling the minority class and combined under-/oversampling of clinical observer diary data.^16,17^

In addition to the standard validity measures, we calculated from the 2×2 contingency tables the balanced accuracy as the average of sensitivity and specificity in percent,^18^ the Matthews correlation coefficient (MCC or Φ coefficient) and the F1-score as the harmonic mean of sensitivity and precision as two test performance measures which are insensitive to dataset imbalances.^19-21^ Of note, Cohen’s κ in the positive range (as in our study) but not in the negative range is strongly correlated to the MCC as a test performance parameter, which is rather insensitive to data imbalance.^19-22^

**SUPPLEMENTARY RESULTS**

**Demographic and clinical characteristics of study cohort.**

55 patients were considered for participation in the study and 47 (85%) of them ended up being included. Based on the aforementioned exclusion criteria, seven participants were excluded. Two were excluded due to MoCA scores below 21 points, two did not properly adhere to the motor diary assessments and three did not adhere to the NMS diary assessments. An additional participant chose to decline further participation in the study following the screening visit. No reasons for their withdrawal were disclosed. The study cohort consisted of 24 (51%) male and 23 (49%) female participants with a median age of 65 (IQR: 58-73). Structured interviews were held to assess types of- motor fluctuations experienced by the participants as displayed in ***Table 1***. The median disease duration was recorded as 10 years (IQR: 8-15 years) with a median symptom duration of 12 years (IQR: 9-17 years) and duration of MF of 61 months (IQR: 34-106 months). A median total levodopa equivalent dose (LED) was calculated to 1,325 mg/day (IQR: 1,025-1,667). Included PD patients had a total median MDS-UDPRS score of 64 (IQR: 52-83), a Hoehn & Yahr stage score of 2.5 (IQR: 2-3), a MoCA score of 27 (IQR: 25-28), NMSS score of 36 (IQR: 29-60), BDI-2 score of 10 (IQR: 4-16), and a PDQ-39 score of 53 (IQR: 33-67). Detailed demographic and clinical data are presented in its entirety in ***Supplementary Table S1***.

**Diary adherence.**

As displayed in ***Supplementary Table S2***, a total of 742 patient-rated hours were recorded in the PD Home diary (hours rated Asleep were excluded from the analysis). We derived 688 hour time periods (92.7% of all waking daytime periods) with complete simultaneous ratings of motor states in patient diaries and NMS diary data. 498 hour periods (99.0%) with complete simultaneous observer ratings of motor states and NMS diary data were recorded. 487 hour periods (96.8% of awake hour periods) were available with all three diary ratings, namely the PD Home diary, the clinical observer motor diary and the NMS diary.

**Diary data on motor and non-motor symptom patterns.**

From the PD home diary ratings, 216 hour time periods out of 742 hourly ratings (or 29.1%) were rated Off, 368 (49.6%) were rated On, and 158 (21.3%) hours were rated On with dyskinesia (***Supplementary Table S2***). The clinical observer diary consisted of 503 simultaneous hours rated by professional clinical observers. Out of these 145 (28.8%) hours were rated motor Off, 178 (35.4%) hours were rated On, and 180 (35.4%) hours were rated On with dyskinesia.

688 waking day hours of complete NMS diary rating were made by participants. Out of these hours, 423 (61.5%) saw simultaneous ratings of one or more NMS. The most commonly occurring NMS were fatigue, pain and inner restlessness at 198 (28.8%), 191 (27.8%) and 165 (24.0%) hours respectively. Hallucinations were only reported for 12 (1.7%) hours. Anxiety and bladder urgency were both only reported for 28 (4.1%) hours each. These were the least frequent patient-rated NMS. The ratings for anxiety, depressive mood, inner restlessness, concentration difficulties and hallucinations were aggregated into psychiatric NMS while excessive sweating, drooling, bladder urgency and dizziness were aggregated into autonomic NMS. Participants reported psychiatric NMS for 222 (32.3%) hours and autonomic NMS for 163 (23.7%) hours.

**Influence of sex on PD Home diary test performance/validity measures.**

Since risk and expression of motor complications, particularly dyskinesia, as well as of several NMS (e.g. depression, anxiety) and presumably their perception largely depend on the sex of the patients,^23-28^ we performed additional exploratory analyses of the influence of sex on the validity of the PD home motor diary. ***Supplementary Figure S1*** shows the overall accuracy of the PD Home motor diary in dependence of simultaneous NMS with respect to the sex of the participants. We did not observe relevant differences of the accuracies between female and male participants. Similar results were obtained for Cohen’s κ vales (***Supplementary Figure S2***) as well as other major test performance measures (accuracy, sensitivity, specificity FPR and FNR; data not shown). Together, there was no indication that sex influences the validity of the PD Home motor diary.

**Data imbalance and PD Home diary test performance/validity measures.**

With class imbalance ratios (IRs) of the clinical outcome measures (IR: number of majority class samples / number of minority class samples) between 1.14 and 4.76 for the clinical observer diary data, our datasets were not or only mildly imbalanced (although there is no general rule for the interpretation of class imbalance in the literature, an IR of 5 or higher is usually considered a moderate to severe imbalance).^16^ However, to address the influence of dataset imbalance on adPMD test performance analyses, we used two approaches: First, calculating additional test performance measures, which are largely independent of dataset imbalance, such as balanced accuracy^18^ or Matthew correlation coefficient (MCC).^19-22^ Secondly, we balanced our datasets using three different approaches prior to calculating various test validity parameters.

***Supplementary Tables S3,S4*** show major test performance measures of the PD Home motor diary for the detection of clinical observer diary motor states with respect to co-occurring NMS and NMS burden (number of simultaneously present NMS). As reported previously, within the positive range of Cohen’s κ, Cohen’s κ is correlated to the MCC as a test performance parameter, which is rather insensitive to data imbalance.^19-22^ Consistently, Cohen’s κ values of the present study (all values were within the positive range) closely corresponded to the respective MCC values (***Supplementary Tables S3,S4***).

We next balanced the test datasets with respect to the three clinical observer-documented motor states as well as to the PD home diary motor states using three different approaches (random undersampling, random oversampling and random combined under-/oversampling).^16,17^ Statistical analyses of balanced datasets in comparison to the original datasets revealed in general no relevant systematic influence of data imbalance in the original datasets with stable values for major performance measures, particularly balanced accuracy and Cohen’s κ (see ***Supplementary Tables S5-S7***).

Together, the major test performance/validity measures sensitivity, specificity, balanced accuracy and Cohen’s κ were not relevantly influenced by the mild degree of dataset imbalance in the present study and thus meaningful for the interpretation of the test performance/validity of the PD Home diary with respect to simultaneous occurring NMS. There was thus no indication that the mild imbalance of the datasets impacted the results on the reported performance measures.

**Times in the 7-meter Timed-Up-and-Go-Test and NMS diary ratings.**

We reported previously that times in the 7-meter Timed-Up-and-Go-Test (7m-TUGT) as a continuous measure of motor performance/dysfunction^29^ displayed a close relationship to motor Off state periods.^2,30^ In contrast, in motor Off state hours from both the PD Home diary and the observer-rated diary, 7m-TUGT results did not display any association with simultaneous NMS occurrence as documented in NMS diaries (***Supplementary Figure S3***). This also applies to the associations of 7m-TUGT results with the occurrence of aggregated NMS (psychiatric NMS, autonomic NMS), pain and fatigue (*P*≥0.05; Mann-Whitney U-test) as well as NMS burden (*P*≥0.05; Jonckheere-Terpstra test). Together, there is no indication that NMS patterns are related to bradykinesia severity during Off state hours.

## **Relationship between 7m-TUGT results and PD Home diary Off state ratings.**

Univariate binary logistic regression of of all hourly diary ratings (independent of NMS diary results) showed significant association of 7m-TUGT results with the likelihood that participants rated motor Off state in the PD Home diary (χ^2^=65.8, *P*<0.001; Nagelkerke R^2^: 18%; correctly classified ratings: 78%): Increasing times in the 7m-TUGT were associated with an increased likelihood of motor Off state ratings (OR: 1.15; 95%CI: 1.11-1.19; *P*<0.001). Corresponding multivariate regression revealed significant associations of both 7m-TUGT times and NMS co-occurrence with motor Off state ratings (χ^2^(2)=84.8, *P*<0.001; Nagelkerke R^2^: 25%; correctly classified ratings: 79%): Increasing 7m-TUGT times were again associated with an increased likelihood of motor Off state ratings (OR: 1.14; 95%CI: 1.09-1.18; *P*<0.001), and Off ratings were 3.80 (95%CI: 2.15-6.70; *P*<0.001) times more likely in NMS^+^ hours compared to NMS^-^ hours.

Consistently, logistic regression plots for NMS^-^ and NMS^+^ hour show that the relationship between 7m-TUGT results and PD Home motor Off state diary ratings differs between NMS^-^ and NMS^+^ hours with higher probability of motor Off state ratings in NMS^+^ as compared to NMS^-^ hours (***Supplementary Figure S3***). In agreement with the absence of any association of NMS occurrence and 7m-TUGT results (see above), the plots do not display major interaction effects between times in the 7m-TUGT and NMS occurrence on participant motor Off state ratings. Similar results were obtained for aggregated NMS co-occurrence and fatigue (see ***Supplementary Table S8***).

**Relationship between 7m-TUGT results and observer-documented motor Off state ratings.**

Univariate binary logistic regression analysis of all hourly diary ratings showed significant effects of times in the 7m-TUGT on the likelihood that the clinical observer simultaneously rates motor Off state (χ^2^=137.5, *P*<0.001; Nagelkerke R^2^: 37%; correctly classified ratings: 80%): Increasing times in 7m-TUGT were associated with an increased likelihood of motor Off state ratings (OR: 1.27; 95%CI: 1.21-1.33; *P*<0.001). Corresponding multivariate regression revealed a significant association 7m-TUGT times and NMS co-occurrence with pbserver-documented motor Off ratings (χ^2^(2)=140.1, *P*<0.001; Nagelkerke R^2^: 38%; correctly classified ratings: 80%): Increasing 7m-TUGT times were again associated with an increased likelihood of motor Off state ratings (OR: 1.26; 95%CI: 1.20-1.32; *P*<0.001), but NMS occurrence did not change the likelihood of observer motor Off ratings (OR:1.94; 95%CI: 0.94-3.39; *P*=0.056).

Consistently, logistic regression plots for NMS^-^ and NMS^+^ hour time periods show that the relationship between 7m-TUGT results and observer-documented motor Off state ratings differs only marginally between NMS^-^ and NMS^+^ hour time periods (***Supplementary Figure S3***; for numeric results, see ***Supplementary Table S8***). The plots do not display major interaction effects between 7m-TUGT times and NMS occurrence on observer-documented motor Off state ratings. In comparison to the corresponding logistic regression plots for PD Home diary Off ratings (***Supplementary Figure S3***), the probability of participant Off state ratings is generally higher in NMS^+^ time periods and approaches the probabilities of observer-documented Off state ratings in NMS^-^ time periods. Notably, the slopes of the logistic regression plots are steeper in observer diary data as compared to PD Home diary data suggesting a sharper demarcation between motor Off and non-Off states by the observer as compared to the participants when using the 7m-TUGT results as the outside criterion.^29^ The same logistic regression analyses obtained for aggregated NMS co-occurrence, fatigue and pain revealed similar results (***Supplementary Table S8***).

Together, simultaneous NMS occurrence increased the probability of motor Off state ratings by the participants but has no relevant effects on participant motor Off ratings when using the 7m-TUGT results as the outside criterion. However, although the correctly classified ratings of the regression models are in general good (≥77% in multivariate models), Nagelkerkes R^2^ values indicate only acceptable amounts of explained variance by the regression models^31^ suggesting that 7m-TUGT has limited predictive value for Off state ratings and/or that there are other – as yet unknown – factors influencing the probability of Off state rating in both the participant the and observer diary.

**SUPPLEMENTARY DISCUSSION**

**Study limitations.**

Firstly, the study cohort consisted of a rather small sized and heterogeneous inpatient cohort from two movement disorder centers, which is however similar to larger cohorts investigating NMS in advanced PD.^32,33^ Moreover, we only recruited PD patients already experiencing motor fluctuations and intentionally excluded patients who screened positive for dementia to ensure proper understanding of motor and NMS states and adherence to the hourly ratings. These aspects could limit generalizability of our results to a greater population including patients with cognitive dysfunction. Moreover, the resulting diary data quantity per participant prevents comparisons of the diary data with patient-specific demographic or clinical characteristics. However, in our previous report using a larger dataset largely acknowledge our present diary-based results by showing on the patient level that depression (BDI) and cognitive dysfunction (MoCA) are independent predictors of PD home diary agreement with clinical observer ratings.^2^

Secondly, motor and NMS were self-reported using diaries including the risk of misunderstandings and misinterpretations of both the practicalities of the diary as well as the definitions of the different motor and non-motor states. To prospectively address these aspects, we used an already introduced NMS diary based on questions from a validated questionnaire (NMSQ).^5^ Further precautions were made as study participants were prior to participation educated on both filling out the diaries and the characteristic features of the included motor and non-motor symptoms. We also used an instructional video to enhance understanding of different motor states and utilized an adopted version of the PD home diary with pictograms.^2^ Another aspect of using paper-based diaries is the issue of diary fatigue.^34,35^ To prevent diary fatigue as well as possible, we selected only eleven key NMS without severity quantification to limit data quality as little as possible due to bothering the patients too much. Furthermore, we here used only data from the first study day and, additionally, patients were frequently reminded to perform the diary ratings by the clinical observer.

Thirdly, we applied clinical observation of motor performance as the outside validation criterion associated with potential limitations and the risk of high inter-rater variability. Although data on inter-rater agreement for clinical observer diary ratings are lacking, relevant variations of the thresholds for the motor states are likely due to differences in the obeservers’ normative experiences (for a detailed discussion, refer to ^30^). We herein used a single-rater approach with one specifically trained and certified clinical observer to exclude potential bias and further validated observer diary responses against the results of a simultaneously assessed 7m-TUGT.^2^ Additional multivariate logistic regression analyses revealed clear effects of the quantitative motor performance measure from the 7m-TUGT but no influences of NMS occurrence ratings on the likelihood of simultanous observer-rated motor Off. Moreover, there are not interaction effects between NMS occurrence and the bradykinesia measure on the probability of observer-rated motor Off state. However, explanatory power of the logistic regression analyses suggests that they explain only an acceptable proportion of variance. There are several potential explanations for this result: First, although there is a clear and reliable relationship of timed motor performance tests with bradykinesia and motor dysfunction in PD,^2,29,36^ times in the 7m-TUGT have limited predictive value for Off state ratings, because 7m-TUGT also measures gait and balance performance. Secondly, NMS as measured by qualitative diary assessment might not sufficient to unfold a predictive value for Off state ratings. Thirdly, there might other – as yet unknown – factors influencing the likelihood of motor Off ratings. Taken together, the exact accuracy of the clinical rater’s motor assessments is yet known, largely due to the current unavailability of other suitable validation criteria for motor diaries.

**Supplementary References**

1. Timpka J, Löhle M, Bremer A, et al. Objective Observer vs. Patient Motor State Assessments Using the PD Home Diary in Advanced Parkinson's Disease. *Front Neurol* 2022; 13: 935664. DOI: 10.3389/fneur.2022.935664.

2. Lohle M, Bremer A, Gandor F, et al. Validation of the PD home diary for assessment of motor fluctuations in advanced Parkinson's disease. *NPJ Parkinsons Dis* 2022; 8: 69. DOI: 10.1038/s41531-022-00331-w.

3. Hauser RA, Russ H, Haeger DA, et al. Patient evaluation of a home diary to assess duration and severity of dyskinesia in Parkinson disease. *Clin Neuropharmacol* 2006; 29: 322-330. DOI: 10.1097/01.WNF.0000229546.81245.7F.

4. Podsiadlo D and Richardson S. The timed "Up & Go": a test of basic functional mobility for frail elderly persons. *J Am Geriatr Soc* 1991; 39: 142-148. DOI: 10.1111/j.1532-5415.1991.tb01616.x.

5. Ossig C, Sippel D, Fauser M, et al. Assessment of Nonmotor Fluctuations Using a Diary in Advanced Parkinson's disease. *J Parkinsons Dis* 2016; 6: 597-607. DOI: 10.3233/JPD-150764.

6. Storch A, Bremer A, Gandor F, et al. Pain Fluctuations in Parkinson's Disease and Their Association with Motor and Non-Motor Fluctuations. *J Parkinsons Dis* 2024; 14: 1451-1468. DOI: 10.3233/JPD-240026.

7. Dalrymple-Alford JC, MacAskill MR, Nakas CT, et al. The MoCA: well-suited screen for cognitive impairment in Parkinson disease. *Neurology* 2010; 75: 1717-1725. DOI: 10.1212/WNL.0b013e3181fc29c9.

8. Hoehn MM and Yahr MD. Parkinsonism: onset, progression and mortality. *Neurology* 1967; 17: 427-442.

9. Goetz CG, Tilley BC, Shaftman SR, et al. Movement Disorder Society-sponsored revision of the Unified Parkinson's Disease Rating Scale (MDS-UPDRS): scale presentation and clinimetric testing results. *Mov Disord* 2008; 23: 2129-2170. DOI: 10.1002/mds.22340.

10. Storch A, Odin P, Trender-Gerhard I, et al. [Non-motor Symptoms Questionnaire and Scale for Parkinson's disease. Cross-cultural adaptation into the German language]. *Der Nervenarzt* 2010; 81: 980-985. DOI: 10.1007/s00115-010-3010-z.

11. Beck AT, Ward CH, Mendelson M, et al. An inventory for measuring depression. *Arch Gen Psychiatry* 1961; 4: 561-571.

12. Jenkinson C, Fitzpatrick R, Peto V, et al. The Parkinson's Disease Questionnaire (PDQ-39): development and validation of a Parkinson's disease summary index score. *Age Ageing* 1997; 26: 353-357.

13. Jost ST, Kaldenbach MA, Antonini A, et al. Levodopa Dose Equivalency in Parkinson's Disease: Updated Systematic Review and Proposals. *Mov Disord* 2023; 38: 1236-1252. DOI: 10.1002/mds.29410.

14. Cicchetti DV. Multiple comparison methods: establishing guidelines for their valid application in neuropsychological research. *J Clin Exp Neuropsychol* 1994; 16: 155-161. DOI: 10.1080/01688639408402625.

15. Tobi H, van den Berg PB and de Jong-van den Berg LT. Small proportions: what to report for confidence intervals? *Pharmacoepidemiol Drug Saf* 2005; 14: 239-247. DOI: 10.1002/pds.1081.

16. Kumar V, Lalotra GS, Sasikala P, et al. Addressing Binary Classification over Class Imbalanced Clinical Datasets Using Computationally Intelligent Techniques. *Healthcare (Basel)* 2022; 10: 1293. DOI: 10.3390/healthcare10071293.

17. Lemaitre A, Nogueira F and Aridas CK. Jmbalanced-learn: A Python Toolbox to Tackle the Curse of Imbalanced Datasets in Machine Learning. *J Machine Learn Research* 2017; 18.

18. Wei Q and Dunbrack RL, Jr. The role of balanced training and testing data sets for binary classifiers in bioinformatics. *PLoS One* 2013; 8: e67863. DOI: 10.1371/journal.pone.0067863.

19. Chicco D, Warrens MJ and Jurman G. The Matthews Correlation Coefficient (MCC) is More Informative Than Cohen’s Kappa and Brier Score in Binary Classification Assessment. *IEEE Access* 2021; 9: 78368-78381.

20. Chicco D and Jurman G. The advantages of the Matthews correlation coefficient (MCC) over F1 score and accuracy in binary classification evaluation. *BMC Genomics* 2020; 21: 6. DOI: 10.1186/s12864-019-6413-7.

21. Boughorbel S, Jarray F and El-Anbari M. Optimal classifier for imbalanced data using Matthews Correlation Coefficient metric. *PLoS One* 2017; 12: e0177678. DOI: 10.1371/journal.pone.0177678.

22. Chicco D, Totsch N and Jurman G. The Matthews correlation coefficient (MCC) is more reliable than balanced accuracy, bookmaker informedness, and markedness in two-class confusion matrix evaluation. *BioData Min* 2021; 14: 13. DOI: 10.1186/s13040-021-00244-z.

23. Hassin-Baer S, Molchadski I, Cohen OS, et al. Gender effect on time to levodopa-induced dyskinesias. *J Neurol* 2011; 258: 2048-2053. DOI: 10.1007/s00415-011-6067-0.

24. Olanow CW, Kieburtz K, Rascol O, et al. Factors predictive of the development of Levodopa-induced dyskinesia and wearing-off in Parkinson's disease. *Mov Disord* 2013; 28: 1064-1071. DOI: 10.1002/mds.25364.

25. Meoni S, Macerollo A and Moro E. Sex differences in movement disorders. *Nat Rev Neurol* 2020; 16: 84-96. DOI: 10.1038/s41582-019-0294-x.

26. Cattaneo C and Pagonabarraga J. Sex Differences in Parkinson's Disease: A Narrative Review. *Neurol Ther* 2025; 14: 57-70. DOI: 10.1007/s40120-024-00687-6.

27. Martinez-Martin P, Falup Pecurariu C, Odin P, et al. Gender-related differences in the burden of non-motor symptoms in Parkinson's disease. *J Neurol* 2012; 259: 1639-1647. DOI: 10.1007/s00415-011-6392-3.

28. Maas BR, Gottgens I, Tijsse Klasen HPS, et al. Age and gender differences in non-motor symptoms in people with Parkinson's disease. *Front Neurol* 2024; 15: 1339716. DOI: 10.3389/fneur.2024.1339716.

29. Morris S, Morris ME and Iansek R. Reliability of measurements obtained with the Timed "Up & Go" test in people with Parkinson disease. *Phys Ther* 2001; 81: 810-818. DOI: 10.1093/ptj/81.2.810.

30. Lohle M, Timpka J, Bremer A, et al. Application of single wrist-wearable accelerometry for objective motor diary assessment in fluctuating Parkinson's disease. *NPJ Digit Med* 2023; 6: 194. DOI: 10.1038/s41746-023-00937-1.

31. Backhaus K, Erichson B, Gensler S, et al. *Multivariate Analysemethoden*. 17th edition ed. Berlin: Springer Gabler, 2023.

32. Storch A, Schneider CB, Wolz M, et al. Nonmotor fluctuations in Parkinson disease: severity and correlation with motor complications. *Neurology* 2013; 80: 800-809. DOI: 10.1212/WNL.0b013e318285c0ed.

33. Stocchi F, Antonini A, Barone P, et al. Early DEtection of wEaring off in Parkinson disease: The DEEP study. *Parkinsonism Relat Disord* 2014; 20: 204-211. DOI: 10.1016/j.parkreldis.2013.10.027.

34. Papapetropoulos SS. Patient diaries as a clinical endpoint in Parkinson's disease clinical trials. *CNS neuroscience & therapeutics* 2012; 18: 380-387. DOI: 10.1111/j.1755-5949.2011.00253.x.

35. Stone AA, Shiffman S, Schwartz JE, et al. Patient non-compliance with paper diaries. *BMJ* 2002; 324: 1193-1194.

36. Ozkan T, Soke F, Erkoc Ataoglu NE, et al. 4-meter walk test in patients with Parkinson's disease: A reliability and validity study. *Gait Posture* 2025; 119: 87-92. DOI: 10.1016/j.gaitpost.2025.02.019.
